# Butyrate ameliorates quinolinic acid–induced cognitive decline in obesity models

**DOI:** 10.1172/JCI154612

**Published:** 2023-02-15

**Authors:** Xing Ge, Mingxuan Zheng, Minmin Hu, Xiaoli Fang, Deqin Geng, Sha Liu, Li Wang, Jun Zhang, Li Guan, Peng Zheng, Yuanyi Xie, Wei Pan, Menglu Zhou, Limian Zhou, Renxian Tang, Kuiyang Zheng, Yinghua Yu, Xu-Feng Huang

**Affiliations:** 1 Jiangsu Key Laboratory of Immunity and Metabolism, Jiangsu International Laboratory of Immunity and Metabolism, Department of Pathogen Biology and Immunology, Xuzhou Medical University, Jiangsu, China.; 2 Department of Neurology, Affiliated Hospital of Xuzhou Medical University, Jiangsu, China.; 3 Affiliated Hospital of Liaoning University of Traditional Chinese Medicine, Shenyang, Liaoning, China.; 4 The Second Affiliated Hospital of Liaoning University of Traditional Chinese Medicine, Shenyang, Liaoning, China.; 5 Illawarra Health and Medical Research Institute (IHMRI) and School of Medical, Indigenous, and Health, University of Wollongong, New South Wales, Australia.

**Keywords:** Aging, Metabolism, Neurodegeneration, Obesity

## Abstract

Obesity is a risk factor for neurodegenerative disease associated with cognitive dysfunction, including Alzheimer’s disease. Low-grade inflammation is common in obesity, but the mechanism between inflammation and cognitive impairment in obesity is unclear. Accumulative evidence shows that quinolinic acid (QA), a neuroinflammatory neurotoxin, is involved in the pathogenesis of neurodegenerative processes. We investigated the role of QA in obesity-induced cognitive impairment and the beneficial effect of butyrate in counteracting impairments of cognition, neural morphology, and signaling. We show that in human obesity, there was a negative relationship between serum QA levels and cognitive function and decreased cortical gray matter. Diet-induced obese mice had increased QA levels in the cortex associated with cognitive impairment. At single-cell resolution, we confirmed that QA impaired neurons, altered the dendritic spine’s intracellular signal, and reduced brain-derived neurotrophic factor (BDNF) levels. Using *Caenorhabditis elegans* models, QA induced dopaminergic and glutamatergic neuron lesions. Importantly, the gut microbiota metabolite butyrate was able to counteract those alterations, including cognitive impairment, neuronal spine loss, and BDNF reduction in both in vivo and in vitro studies. Finally, we show that butyrate prevented QA-induced BDNF reductions by epigenetic enhancement of H3K18ac at BDNF promoters. These findings suggest that increased QA is associated with cognitive decline in obesity and that butyrate alleviates neurodegeneration.

## Graphical abstract

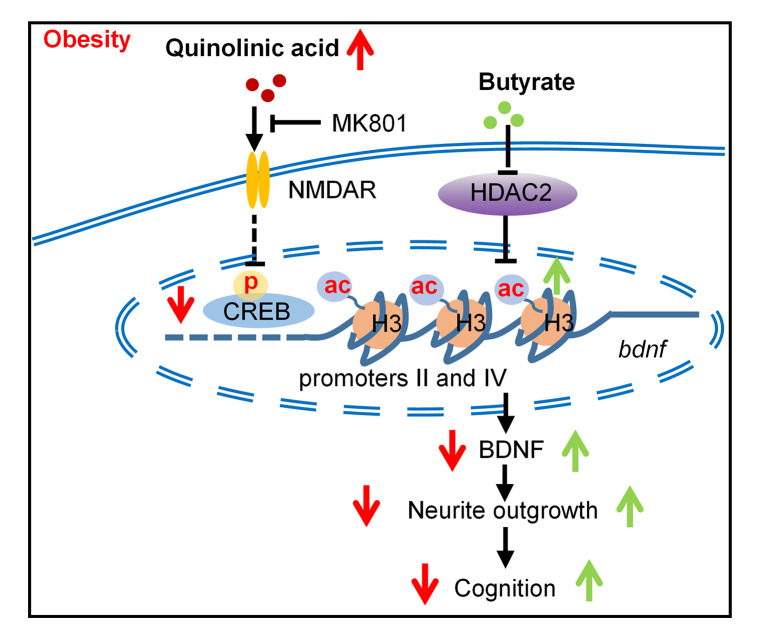

## Introduction

Obesity is a growing health concern that has increased in prevalence worldwide in the past 50 years, reaching pandemic levels (1). Obesity is also associated with cognitive dysfunction and is considered a risk factor for Alzheimer’s disease (AD) (2, 3). Accumulating research reveals a low-grade inflammatory response (activation of microglia and monocyte lineage cells) in the peripheral and central nervous systems in obesity (4). Growing evidence implicates immune cell–mediated tissue inflammation as an important mechanism linking obesity to cognition decline (5). However, the mechanisms by which obesity-associated inflammation leads to cognitive impairment remain to be investigated. Quinolinic acid (QA), an endogenous neurotoxin, is excessively secreted by microglia or monocyte lineage cells during inflammation (6). Research shows that the expression of QA-related enzyme genes is increased in adipose tissue of individuals with obesity (7). Intracranial injection of QA impairs spatial learning and memory in rats (8). Few studies, however, have investigated QA’s role and cellular mechanism underlying cognitive decline in obesity.

Generating and maintaining proper neurite outgrowth and arborization are critical for normal synaptic ultrastructure, neural architecture, and cognitive function (9, 10). Imaging studies suggest that obesity is associated with neural atrophy in clinical studies (11, 12) and synapse loss in animal studies (13), although the findings are inconsistent (14). QA as a neurotoxin is involved in alterations of synaptic function and plasticity in rodents (15) and in the pathogenesis of neurodegenerative diseases, including AD (16). Intracranial injection of QA into mice diminishes the level of brain-derived neurotrophic factor (BDNF) in the cortex, which is important for synaptic morphology and cognition (17). However, the molecular mechanism of QA in the regulation of synaptic ultrastructure and BDNF expression requires further elucidation in obesity. Furthermore, aberrant histone modifications at the BDNF promoters and abnormal BDNF levels have been linked to neurodegenerative disorders (18, 19), indicating that targeting the epigenetic regulation of BDNF would shed light on future therapeutic strategies.

Butyrate is one of the short-chain fatty acids (SCFAs), metabolites produced in the colon by bacterial fermentation of dietary fibers (20). Butyrate is the most effective inhibitor of histone deacetylases (HDACs), with 80% inhibitory efficiency (21), and is the SCFA most abundantly uptaken by the brain (22) compared with other SCFAs, including acetate and propionate. Previously, it was reported that butyrate improved memory in AD mouse models (23); however, the underlying cellular and molecular mechanisms were not investigated. Moreover, decreased concentrations of butyrate and butyrate-producing bacteria are observed in the feces of individuals with obese (24, 25), indicating that butyrate may prevent cognitive decline in obesity. Given its potential functional and epigenetic effects, we hypothesize that butyrate protects against neurotoxin QA–induced cognitive decline through mechanisms of epigenetic regulation of BDNF and promotion of neurite outgrowth.

Here, we report that QA levels markedly increased in the serum of individuals with obesity and in the frontal cortical brain regions of diet-induced obese mice and found that QA levels in both humans and mice were significantly correlated with a decline in cognition. Consistently, we found reduced gray matter volume (GMV) in the frontal, parietal, and temporal lobes of individuals with obesity, as revealed by MRI analysis, and observed impaired synaptogenesis with reduced dendritic arborization, lower spine density, and altered spine morphology in the frontal cortex of obese mice. Importantly, butyrate prevented cognitive impairment in obese mice, and transgenic *Caenorhabditis elegans* exposed to QA, along with improvement of damaged dopaminergic and glutamatergic neurons. Furthermore, butyrate prevented neurite impairment in obese mice and QA-induced dendritic spine loss in SH-SY5Y and primary cortical neurons. We found that the molecular mechanism underlying the improved cognition was attributable to butyrate, which acted as an HDAC2 inhibitor to promote histone H3K18 hyperacetylation and epigenetic regulation of the BDNF promoter regions PII and PIV to increase BDNF expression. These findings collectively suggest that, in humans, rodents, *C. elegans*, and in vitro cell models, excessive QA was associated with cognitive decline and neurite lesions related to obesity, whereas butyrate alleviated the cognitive impairment induced by elevated QA levels or obesity.

## Results

### Serum QA levels are increased and significantly associated with altered cognition in individuals with obesity with or without diabetes.

**Figure 1:**
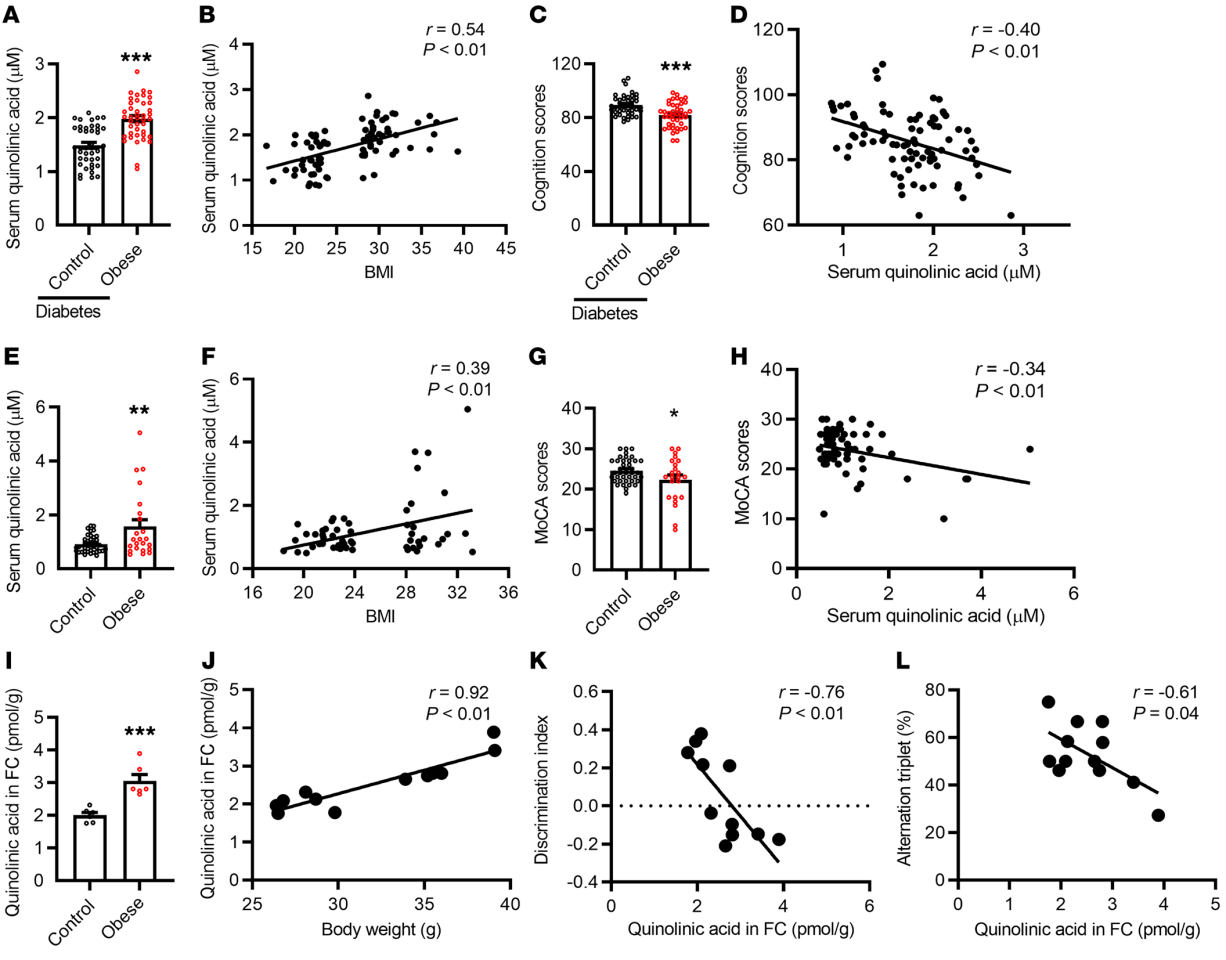
Serum QA is increased and significantly associated with altered cognition in individuals with obesity and in obese mice. (**A**) Serum QA levels were increased in individuals with obesity with type 2 diabetes. (**B**) QA levels were significantly positively correlated with BMI. (**C**) The RBANS total cognition score was lower in individuals with obesity. (**D**) The total cognition score was negatively correlated with serum QA levels. (**E**) Serum QA levels were increased in individuals with obesity who did not have type 2 diabetes. (**F**) QA levels were positively correlated with BMI. (**G**) The MoCA cognition score was lower in individuals with obesity. (**H**) The MoCA score was negatively correlated with serum QA levels. (**I**) QA levels were significantly increased in the frontal cortex of obese mice. (**J**) QA levels in the frontal cortex were highly correlated with body weight. (**K**) Frontal cortical QA concentrations were negatively correlated with the discrimination index in temporal order memory tests. (**L**) The frontal cortical QA concentrations were negatively correlated alternation triplet in Y maze tests. FC, frontal cortex. *P < 0.05, **P < 0.01, and ***P < 0.001 versus the control group, by 2-tailed Student’s *t* test.

Obesity is associated with systemic and neuroinflammation, such as activation of macrophages and microglia (4), which promote the secretion of QA (6). The neuroinflammatory product QA can be toxic and is associated with neurodegenerative processes in neurodegenerative diseases (16). We measured serum QA concentrations and cognitive function in individuals with obesity. In the individuals with type 2 diabetes, we found that QA levels were significantly higher in the serum of those with obesity than in those who were not obese (*P* < 0.01; Figure 1A). QA levels were significantly positively correlated with BMI (*r* = 0.54, *P* < 0.01; Figure 1B) and fasting insulin levels (*r* = 0.32, *P* = 0.02; Supplemental Table 3; supplemental material available online with this article; https://doi.org/10.1172/JCI154612DS1). The total cognition score for the Repeatable Battery for the Assessment of Neuropsychological Status (RBANS) was lower in individuals with obesity (*P* < 0.01; Figure 1C) and negatively correlated with serum QA levels (*r* = –0.40, *P* < 0.01; Figure 1D). Furthermore, individuals with obesity had a lower attention index (*P* < 0.01) than did nonobese individuals (Supplemental Figure 1A), but this was not the case for the delayed memory index, language index, visuospatial and constructional abilities index, and immediate memory index (Supplemental Figure 1, B–E). Serum QA levels were negatively correlated with delayed memory (*r* = –0.31, *P* < 0.01; Supplemental Figure 1F) and attention (*r* = –0.19, *P* < 0.08 in statistic trend; Supplemental Figure 1G), but not with learning, visuospatial and constructional abilities, or immediate memory indices (Supplemental Figure 1, H–J). In the nondiabetic population, serum QA levels were significantly higher in the individuals with obesity than nonobese individuals (*P* < 0.01; Figure 1E). Furthermore, QA levels were positively correlated with BMI (*r* = 0.39, *P* < 0.01; Figure 1F and Supplemental Table 3). The Montreal Cognitive Assessment (MoCA) score was lower for individuals with obesity (*P* < 0.05; Figure 1G) and negatively correlated with serum QA levels (*r* = –0.34, *P* < 0.01; Figure 1H). Thus, an overproduction of QA in obesity may contribute to cognitive decline.

### The cortical QA is increased and significantly associated with reduced cognition in obese mice.

We found that the QA levels were significantly increased in the frontal cortex of high-fat diet–induced obese mice compared with mice on a lab chow diet (*P* < 0.01; Figure 1I). In addition, QA levels in the frontal cortex were positively correlated with body weight (*r* = 0.92, *P* < 0.01; Figure 1J). The frontal cortical QA concentrations were negatively correlated with the cognitive index, including the discrimination index (*r* = –0.76, *P* < 0.01) and the alternation triplet (*r* = –0.61, *P* = 0.04) in temporal order memory and Y maze tests, respectively (Figure 1, K and L). The discrimination index and alternation triplet in temporal order memory and Y maze scores were significantly decreased for obese mice compared with control mice (Supplemental Figure 2, A and B). Obese mice had higher subcutaneous and epididymis fat mass, glucose intolerance, increased total cholesterol, and high- and low-density lipoprotein concentrations (Supplemental Table 4).

### Cortical volume and neurites are altered in individuals with obesity and obese mice.

**Figure 2:**
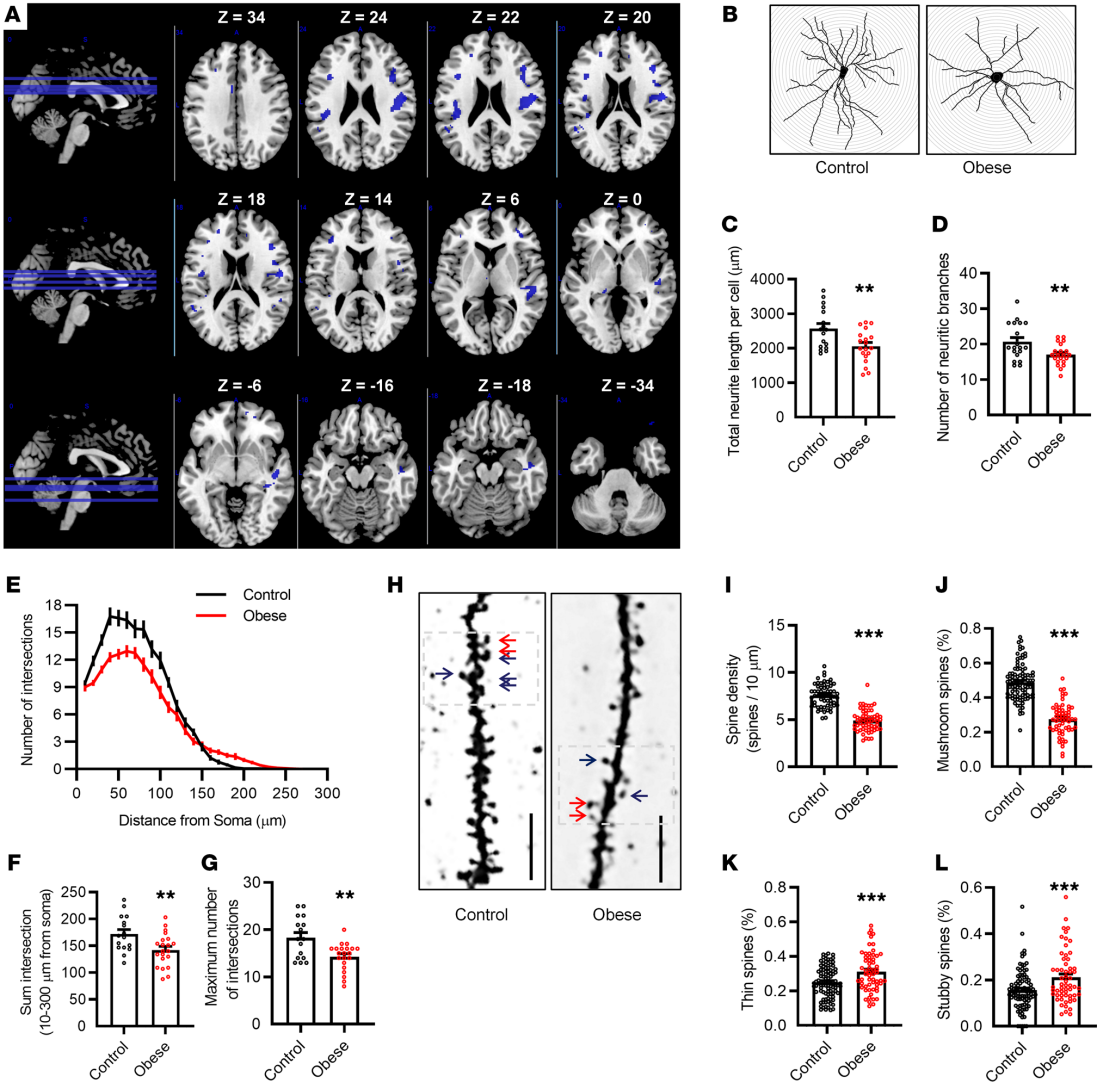
Cortical volume and neuronal morphology are changed in individuals with obesity and obese mice. (**A**) Statistical maps show regions (blue) with reduced GMV in individuals in the obese group as captured by 3D-T1 axial and sagittal slice imaging (MNI T1 template available in MRIcron software). The MRI study included 19 individuals with obesity and 21 age- and sex-matched, nonobese controls. (**B**) Representative reconstructions of Golgi-Cox–stained neurons in the frontal cortex of obese and control mice. (**C**) The total neurite length per cell was decreased in the frontal cortex of obese mice. (**D**) The number of neurite branches was decreased in the frontal cortex of obese mice. (**E**) Neurite morphology was profiled by Sholl analysis. (**F** and **G**) The sum intersection (**F**) and maximum number of intersections (**G**) were calculated by Sholl analysis. (**H**) Synaptic spines were stained with Golgi silver. Blue arrows show mushroom spines. Red arrows show thin spines. (**I**) Spine density, as determined by the number of spines per 10 μm. (**J**–**L**) Ratio of mushroom-like spines (**J**), thin spines (**K**), and stubby spines (**L**). Data indicate the mean ± SEM. *n* = 4 mice/group for Golgi staining. **P < 0.01 and ***P < 0.001 versus the control group, by 2-tailed Student’s *t* test.

We found that individuals with obesity had significant reductions in GMV primarily in the cortical regions (Table 1 and Figure 2A), which include the middle frontal gyrus (left and right), the inferior frontal gyrus (left), the frontal subgyrus (right), the medial frontal gyrus (left), the postcentral parietal gyrus (right), the inferior parietal lobules (left), the superior temporal gyrus (right), the middle temporal gyrus (right), the temporal subgyrus (right), the cingulate gyrus (left), and the insula cortex (left and right). The trend appeared from the frontal lobe, extending backward to the parietal lobe and downward to the temporal lobes and insular cortex. The left thalamus was also reduced in volume. With Golgi silver staining in the frontal cortex of obese mice (Figure 2B and Supplemental Figure 2C), we observed a significant decrease in the total neurite length per cell (*P* < 0.01; Figure 2C) and the number of neurite branches (*P* < 0.01; Figure 2D), with no change in the average neurite length per branch in the frontal cortex (*P* = 0.11; Supplemental Figure 2D). Furthermore, Sholl analysis showed that mice in the obese group had reduced dendritic arborization and complexity in the frontal cortex (Figure 2E) as a result of a decrease in the sum and maximum number of dendritic intersections (*P* < 0.01; *P* < 0.01; Figure 2, F and G), but no alteration in the position of the most complicated dendritic structure with a similar distance of the maximum intersections from the soma (*P* = 0.28; Supplemental Figure 2E). In addition, obesity affected synaptic spine morphology (Figure 2H) by decreasing spine density (*P* < 0.01; Figure 2I), decreasing the ratio of mushroom-like spines (*P* < 0.01; Figure 2J), increasing the ratio of thin spines (*P* < 0.01; Figure 2K), and increasing the ratio of stubby spines (*P* < 0.01; Figure 2L). Therefore, chronic obesity may cause structural and neuronal morphology changes in the brain.

### Butyrate prevents altered cognitive function and neurite impairment in obese mice.

**Figure 3:**
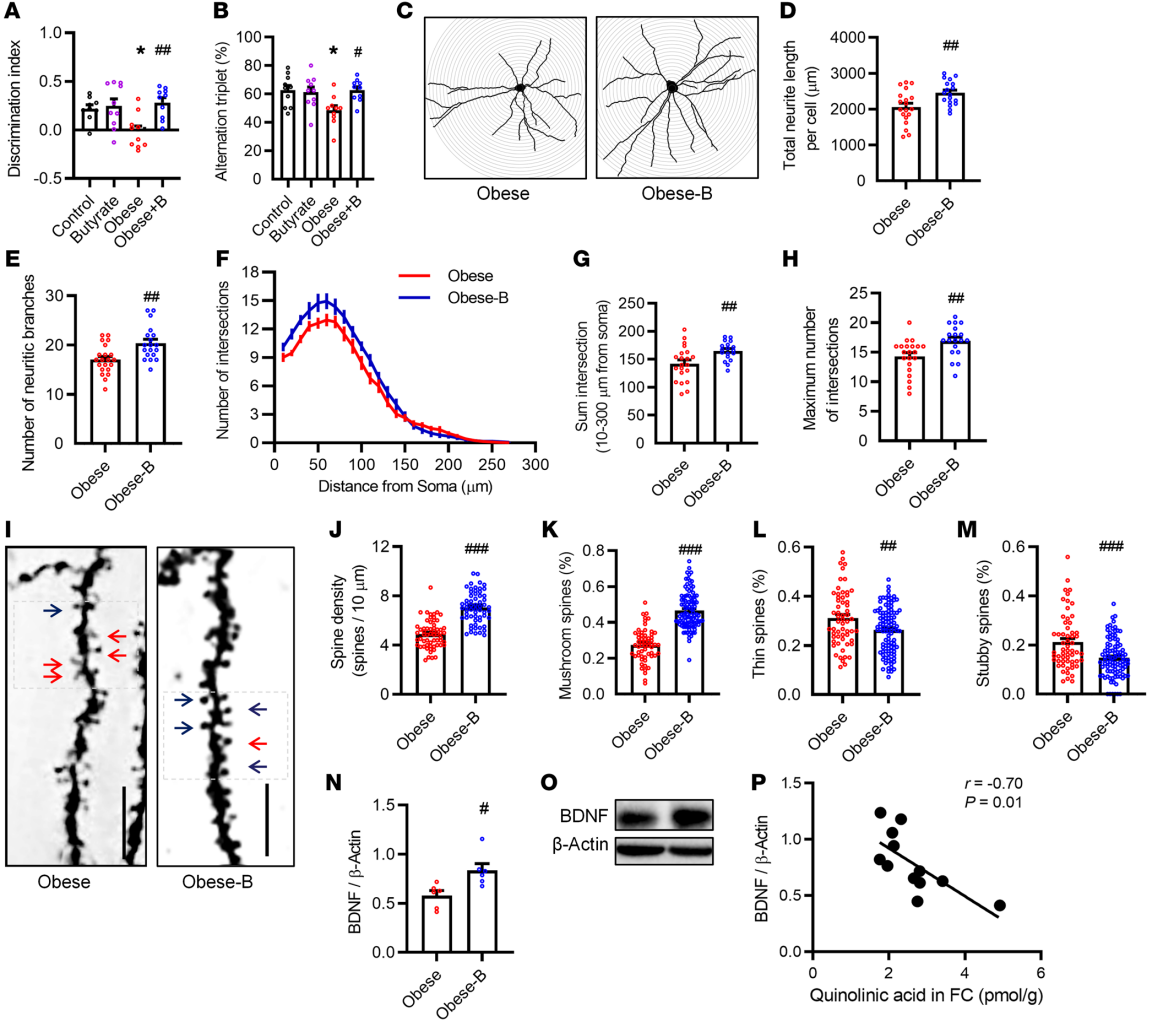
Butyrate prevents altered cognitive function and neurite impairment in obese mice. (**A** and **B**) Butyrate prevented high-fat diet–induced cognitive decline tested by temporal order experiments (**A**) and Y-maze experiments (**B**) (*n* = 10). *P < 0.05 versus the control group. ^#^*P* < 0.05 and ^##^*P* < 0.01 versus the obese group, by 1-way ANOVA with Tukey’s multiple-comparison test. (**C**) Representative reconstructions of Golgi-Cox–stained neurons in the frontal cortex of obese mice. (**D** and **E**) Butyrate supplementation increased the total neurite length per cell (**D**) and the number of neurite branches (**E**) in the frontal cortex of obese mice. (**F**) Neurite morphology was profiled by Sholl analysis. (**G** and **H**) The sum intersection (**G**) and maximum number of intersections (**H**) were calculated by Sholl analysis. (**I**) Synaptic spines were stained with Golgi silver. Blue arrows show mushroom spines; red arrows show thin spines. (**J**) Spine density, as determined by the number of spines per 10 μm. (**K**–**M**) Ratio of mushroom-like spines (**K**), thin spines (**L**), and stubby spines (**M**). (**N** and **O**) Butyrate increased BDNF expression in the cortex of obese mice. (**P**) BDNF expression was associated with QA levels in the frontal cortex. Data in **P** were analyzed by Pearson’s correlation. Data indicate the mean ± SEM. *n* = 4 mice/group for Golgi staining. ^#^*P* < 0.05, ^##^*P* < 0.01, and ^###^*P* < 0.001, by 2-tailed Student’s *t* test (**D**, **E**, **G**, **H**, and **J**–**N**).

The gut microbiome metabolite butyrate can enter the brain via the circulation and improve brain function and neurite outgrowth (26, 27). We observed that butyrate supplementation prevented a decline in the discrimination index among obese mice in the temporal order memory test (*P* < 0.01; Figure 3A). The difference in cognition was not considered to be due to the general activity variation because the total exploration time with the objects during the test phases was similar (Supplemental Figure 2F). In the Y maze spontaneous alternation tests, the alternation triplet was increased in obese mice with butyrate supplementation compared with the obese control mice (*P* < 0.05; Figure 3B), suggesting an improvement in spatial working memory. In addition, butyrate decreased body weight, subcutaneous fat mass, and fasting insulin levels in obese mice (Supplemental Table 5). Furthermore, using body weight as a confounding covariate to eliminate the effect of body weight, butyrate treatment still significantly increased the discrimination index (*F* = 9.06, *P* < 0.01), but not the alternation triplet (*F* = 3.90, *P* = 0.07, Supplemental Table 6).

Butyrate supplementation prevented a decrease in total neurite length per cell (*P* < 0.01; Figure 3, C and D) and in the number of neurite branches in obese mice (*P* < 0.01; Figure 3, C and E). Butyrate supplementation also prevented a decrease in dendritic arborization and complexity in obese mice (Figure 3F), with an increased sum and maximum number of dendritic intersections (*P* < 0.01; *P* < 0.01; Figure 3, G and H). We observed protective effects of butyrate on synaptic spine morphology (Figure 3I) and spine density (*P* < 0.01; Figure 3J) in obese mice. Butyrate prevented a decrease in the percentage of the mushroom-like spines (*P* < 0.01; Figure 3K) and an increase in the percentage of thin spines (*P* < 0.01; Figure 3L) and stubby spines (*P* < 0.01; Figure 3M) induced by obesity. Furthermore, butyrate prevented a reduction of BDNF in the frontal cortex of obese mice (*P* = 0.01; Figure 3, N and O, and Supplemental Figure 2, G and H). We found a negative association between BDNF and QA levels in the frontal cortex (*r* = –0.70, *P* = 0.01; Figure 3P).

### Butyrate prevents QA-induced cognitive dysfunction in C. elegans.

**Figure 4:**
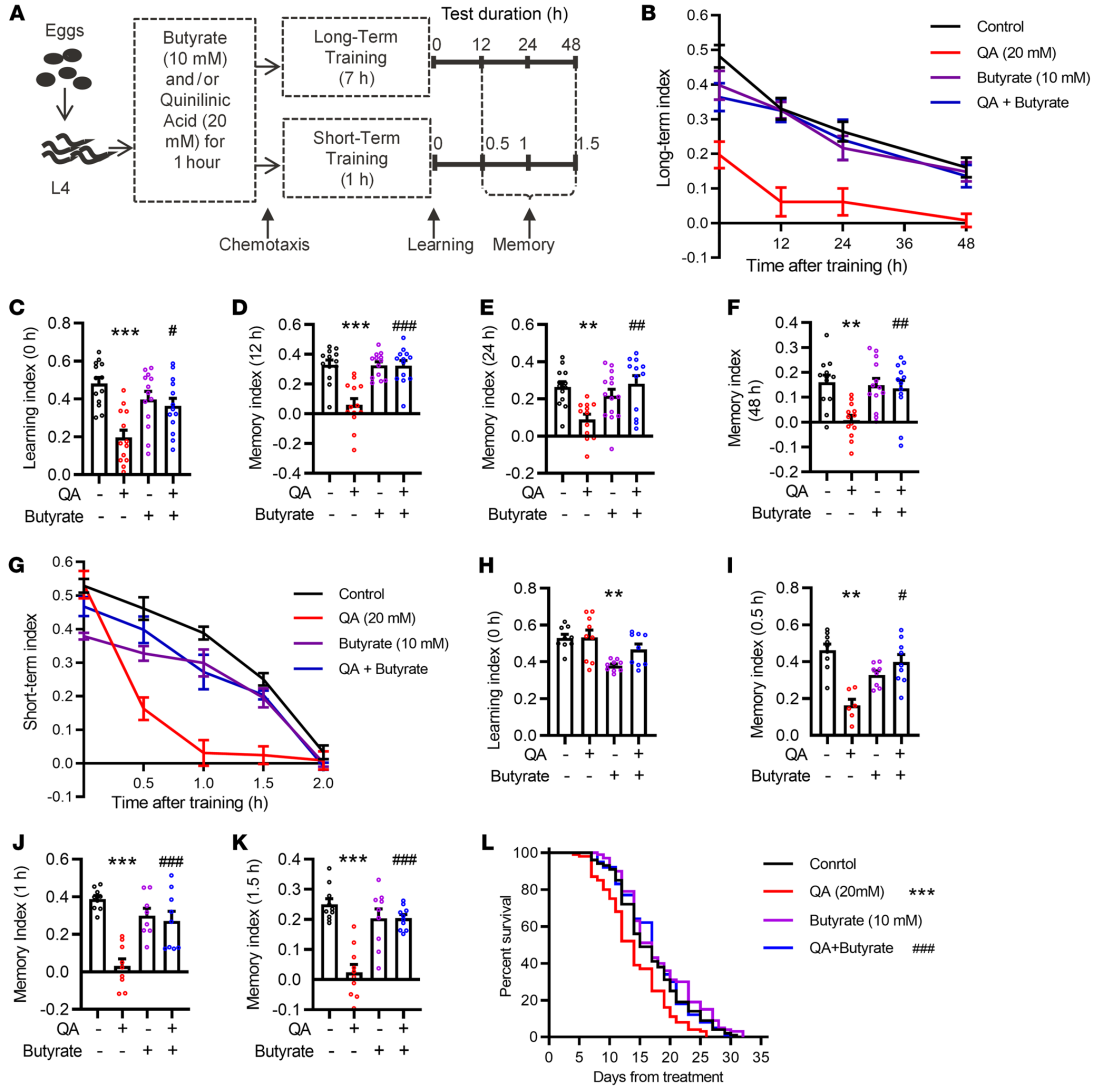
QA-impaired cognitive function is prevented by butyrate in *C.* *elegans*, N2. (**A**) Experimental procedure for learning and memory of *C. elegans*. (**B**) Long-term learning and memory index for *C. elegans* after treatment with QA and/or butyrate. (**C**) Long-term learning index after treatment with QA and butyrate. (**D**–**F**) Long-term memory index for *C. elegans* after treatment with QA and/or butyrate at 12 hours (**D**), 24 hours (**E**), and 48 hours (**F**). (**G**) Short-term learning and memory index for *C. elegans* after treatment with QA and/or butyrate. (**H**) Short-term learning index for *C. elegans* after treatment with QA and/or butyrate. (**I**–**K**) Short-term memory index after treatment with QA and/or butyrate at 0.5 hours (**I**), 1 hour (**J**), and 1.5 hours (**K**). *n* = 5 independent experiments performed in triplicate. Data indicate the mean ± SEM. **P < 0.01 and ***P < 0.001 versus the control group; ^#^*P* < 0.05, ^##^*P* < 0.01, and ^###^*P* < 0.001 versus the QA group, by 1-way ANOVA with Tukey’s multiple-comparison test. (**L**) Butyrate reversed the QA-shortened lifespan (*n* = 125 experiments run in triplicate). Survival data were analyzed by log-rank (Mantel-Cox) test and multiple comparisons of survival data followed Bonferroni’s correction.

Given the above findings that higher QA levels were associated with cognitive decline in obesity and that butyrate had beneficial effects, we used *C. elegans* as a neurodegeneration model to determine whether QA induces cognitive impairment and whether butyrate would prevent the possible deleterious effects of QA (Figure 4A). In the present study, butyrate prevented declines in long-term learning and memory in *C. elegans* following exposure to QA (Figure 4, B–F). The learning index was significantly improved after butyrate treatment in the animals exposed to QA (*P* < 0.05; at 0 hour in Figure 4, B and C). The decline in memory indices was also prevented after butyrate treatment at 12, 24, and 48 hours (all *P* < 0.01; Figure 4, D–F). In the short-term learning and memory test, QA did not affect the learning index (at 0 hour in Figure 4, G and H), but reduced the memory index (Figure 4G, slope, and Figure 4, I–K). Butyrate reversed the QA-induced reduction in the short-term memory index at 0.5, 1, and 1.5 hours (all *P* < 0.05; Figure 4, I–K). Aging-associated cognitive decline is linked to general health deterioration. We observed that QA shortened lifespan and reduced body bends and pharyngeal pumping rates (Figure 4L and Supplemental Figure 3, A–F). Butyrate treatment prevented deleterious effects of QA on *C. elegans* lifespan and body bends (Figure 4L and Supplemental Figure 3, A–E), but not on pharyngeal pumping rates (Supplemental Figure 3F).

### Butyrate prevents QA-induced dopaminergic and glutamatergic neuronal damage in C. elegans.

**Figure 5:**
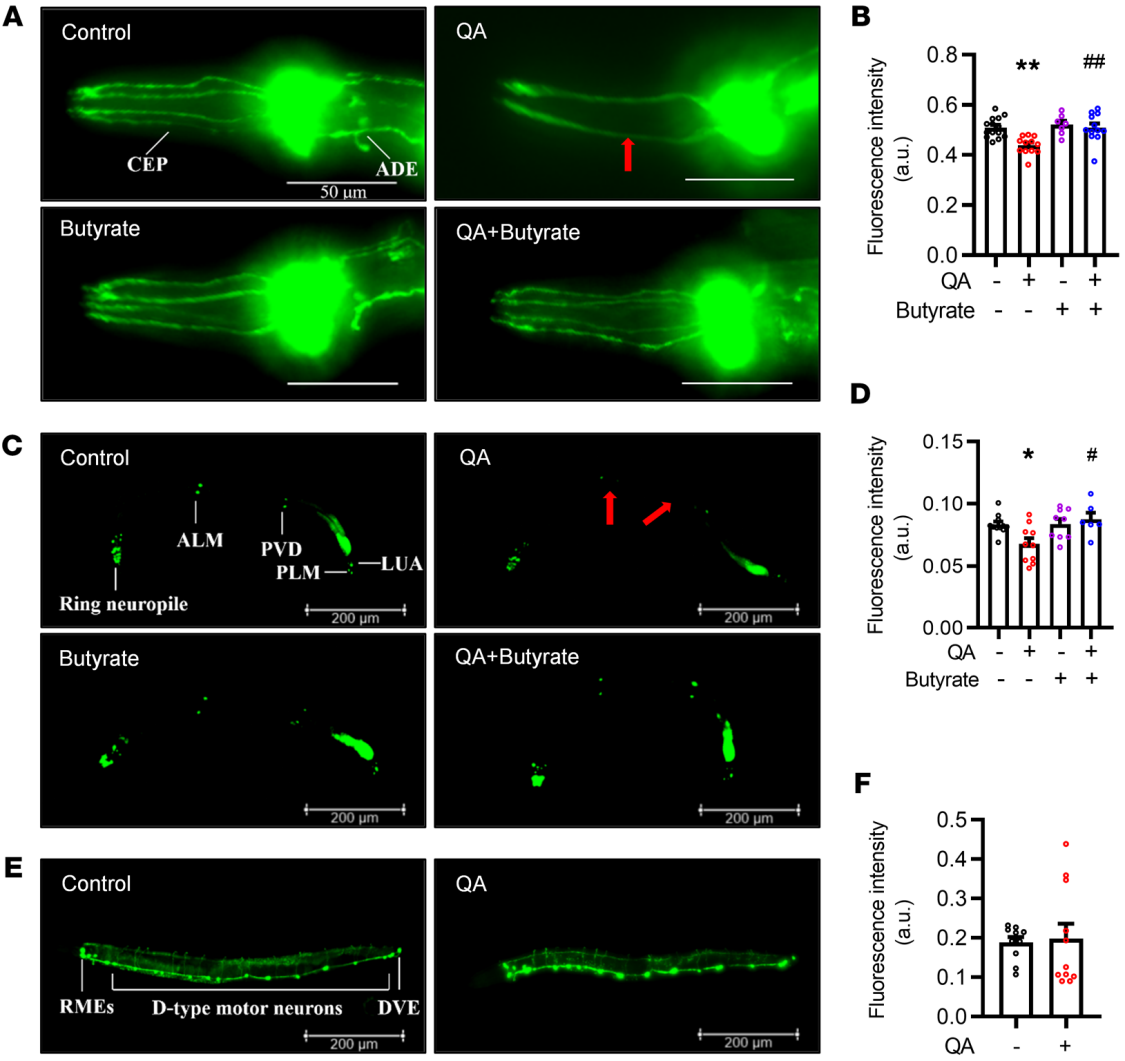
QA damage of dopaminergic and glutamatergic neurons is prevented by butyrate in *C.* *elegans*. Transgenic *C. elegans* strains, including BZ555 (dat-1p::GFP), EG1285 [unc-47p::GFP+lin-15(+)], and DA1240 [eat-4::GFP+lin-15(+)], were used to visualize dopaminergic, glutamatergic, and GABAergic neurons. Worms were exposed to 20 mM QA and/or 10 mM butyrate for 1 hour at L4 stag and then treated with butyrate (10 mM) for 24 hours. Fluorescence images were taken to measure morphology and fluorescence intensity. (**A**) Dopamine neurons were visualized by expression of dat-1p::GFP. Red arrow shows the loss of dendrites in 2 pairs of CEP neurons. Scale bars: 50 μm. ADE, anterior deirid neuron. (**B**) The fluorescence intensity of dopaminergic neurons was significantly different after treatment with QA and/or butyrate. (**C**) Glutamatergic neurons labeled with eat-4::GFP in the control worm. Red arrows show neuronal loss in ALM and PVD neurons. Scale bars: 200 μm. PLM, posterior lateral microtubule neuron; LUA, lumbar ganglion interneuron. (**D**) The fluorescence intensity of glutamatergic neurons was significantly different after exposure to QA and/or butyrate. (**E**) GABAergic neurons were visualized by expression of unc-47::GFP. Scale bars: 200 μm. RMEs, ring motor neuron E; DVE, dorsorectal ventral process E. (**F**) No change in fluorescence intensity of GABAergic neurons was observed after QA treatment (*P* = 0.76). Data indicate the mean ± SEM. *n* = 10 independent experiments performed in triplicate. *P < 0.05 and **P < 0.01 versus the control group; ^#^*P* < 0.05 and ^##^*P* < 0.01 versus the QA group, by 1-way ANOVA with Tukey’s multiple-comparison test.

Next, to investigate the cellular mechanism of butyrate in the prevention of cognitive dysfunction induced by QA, we examined their effects on dopaminergic, glutamatergic, and GABA neurons, which are important for learning and memory (28, 29). We observed that QA reduced the dendrites of dopaminergic neurons (cephalic sensilla [CEP] neurons, *P* < 0.01; Figure 5, A and B), while butyrate ameliorated the loss of dendrites induced by QA. Using the transgenic nematode strain DA1240, with glutamatergic neurons labeled with excitatory amino acid transporter 4::GFP (eat-4::GFP), we found that butyrate prevented the loss of the anterior lateral microtubule (ALM) neuron and the posterior ventral process D (PVD) neuron (Figure 5C) and the reduction of glutamate fluorescence intensity induced by QA (*P* < 0.05; Figure 5D). However, QA did not affect the morphology or fluorescence intensity of GABAergic neurons in the transgenic nematode strain EG1285 with fluorescently labeled GABA neurons (*P* = 0.82; Figure 5, E and F). These results suggest that QA could damage dopaminergic and glutamatergic neurons, but not GABA neurons, in *C. elegans*, whereas butyrate ameliorated QA-induced neurotoxicity.

### Butyrate prevents QA-induced dendritic spine loss in SH-SY5Y and primary cortical neurons.

**Figure 6:**
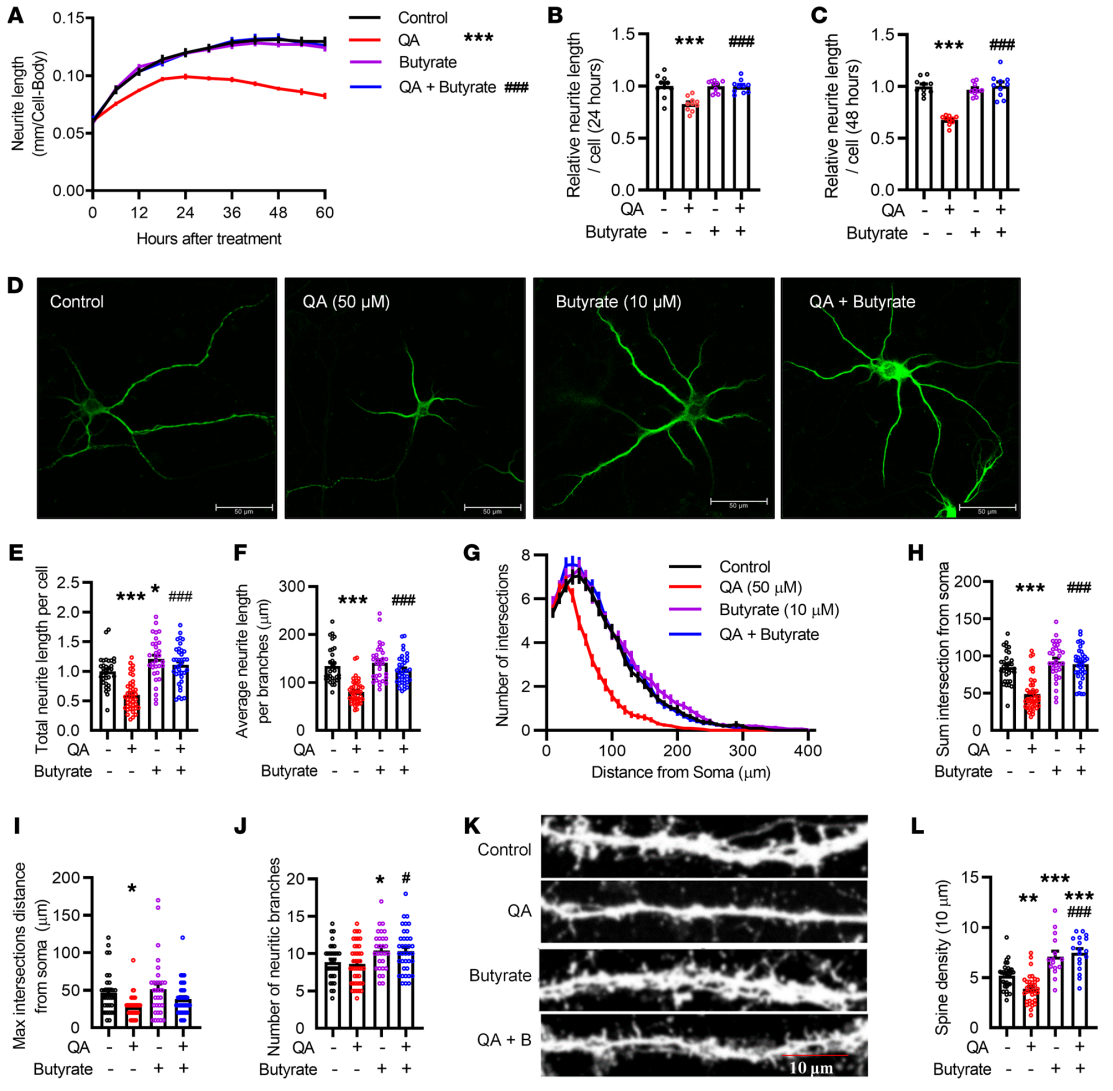
QA-induced shorter neurite outgrowth and synaptic spine loss were prevented by butyrate in SH-SY5Y cells and primary cortical neurons. (**A**) Neurite length of SH-SY5Y cells after treatment with QA (50 μM) and/or butyrate (10 μM) (*n* = 10 experiments run in triplicate). Data were analyzed by repeated-measures, 2-way ANOVA with Tukey’s multiple-comparison test. (**B** and **C**) Relative neurite length of SH-SY5Y cells after treatment for 24 hours (**B**) and 48 hours (**C**). (**D**) Primary frontal cortical neurons (DIV 7) were treated with QA (50 μM) and/or butyrate (10 μM) for 24 hours, stained with MAP2 antibody, and imaged with an immunofluorescence confocal microscope. Scale bars: 50 μm. (**E** and **F**) Neurite length/cell (**E**) and neurite length/branch (**F**) were analyzed by NeuronJ software. (**G**) The number of intersections for every 10 μm length of soma was quantified by Sholl analysis. (**H** and **I**) The sum intersection (**H**) and maximum (Max) intersection distance (**I**) were calculated in the Sholl analysis. (**J**) The number of neurite branches was analyzed by NeuronJ software. (**K**) Synaptic spines of primary frontal cortical neurons (DIV 17) were stained with Alexa Fluor 568 phalloidin and imaged with the immunofluorescence confocal microscope. Scale bar: 10 μm. (**L**) Butyrate prevented QA-induced spine reductions in primary frontal cortical neurons (*n* = 10 experiments run in triplicate). Data indicate the mean ± SEM. *P < 0.05, **P < 0.01, and ***P < 0.001 versus the control group; ^#^*P* < 0.05 and ^###^*P* < 0.001 versus the QA group, by 1-way ANOVA with Tukey’s multiple-comparison test.

Neural connectivity and synaptogenesis are important for cognitive function (30). The doses and time effects of QA and butyrate were studied in real time using IncuCyte quantitative analysis. The neurite length responded to both the doses and durations of the treatments in SH-SY5Y neuronal cells (Supplemental Figure 4, A–F). Butyrate effectively prevented neurite lesions, whereas butyrate alone did not change neurite length in SH-SY5Y neuronal cells (Figure 6, A–C, and Supplemental Figure 4G). Furthermore, in primary mouse frontal cortical neurons, butyrate prevented QA-induced neurite lesions (Figure 6D and Supplemental Figure 4H), as determined by the total neurite length per cell (Figure 6E), the average length per branch (Figure 6F), and arborization (Figure 6, G and H), but butyrate did not affect the distance of the maximum intersections from the soma, the position of the most complicated dendritic structure (Figure 6I). In addition, QA did not affect the number of neurite branches; however, butyrate treatment increased the number of neurite branches regardless of whether QA was administered (Figure 6J). Moreover, butyrate prevented the loss of dendritic spines induced by QA in primary neurons stained with Alexa Fluor 568 phalloidin (*P* < 0.01; Figure 6, K and L). In addition, cell viability was not affected by butyrate (10 μM) or QA (50 μM) (Supplemental Figure 4I). Overall, these results suggest that butyrate prevented QA-impaired neurite outgrowth and dendritic spines in neurons.

### Butyrate prevents QA-induced BDNF reduction by epigenetic enhancement of H3K18ac at BDNF promoters.

**Figure 7:**
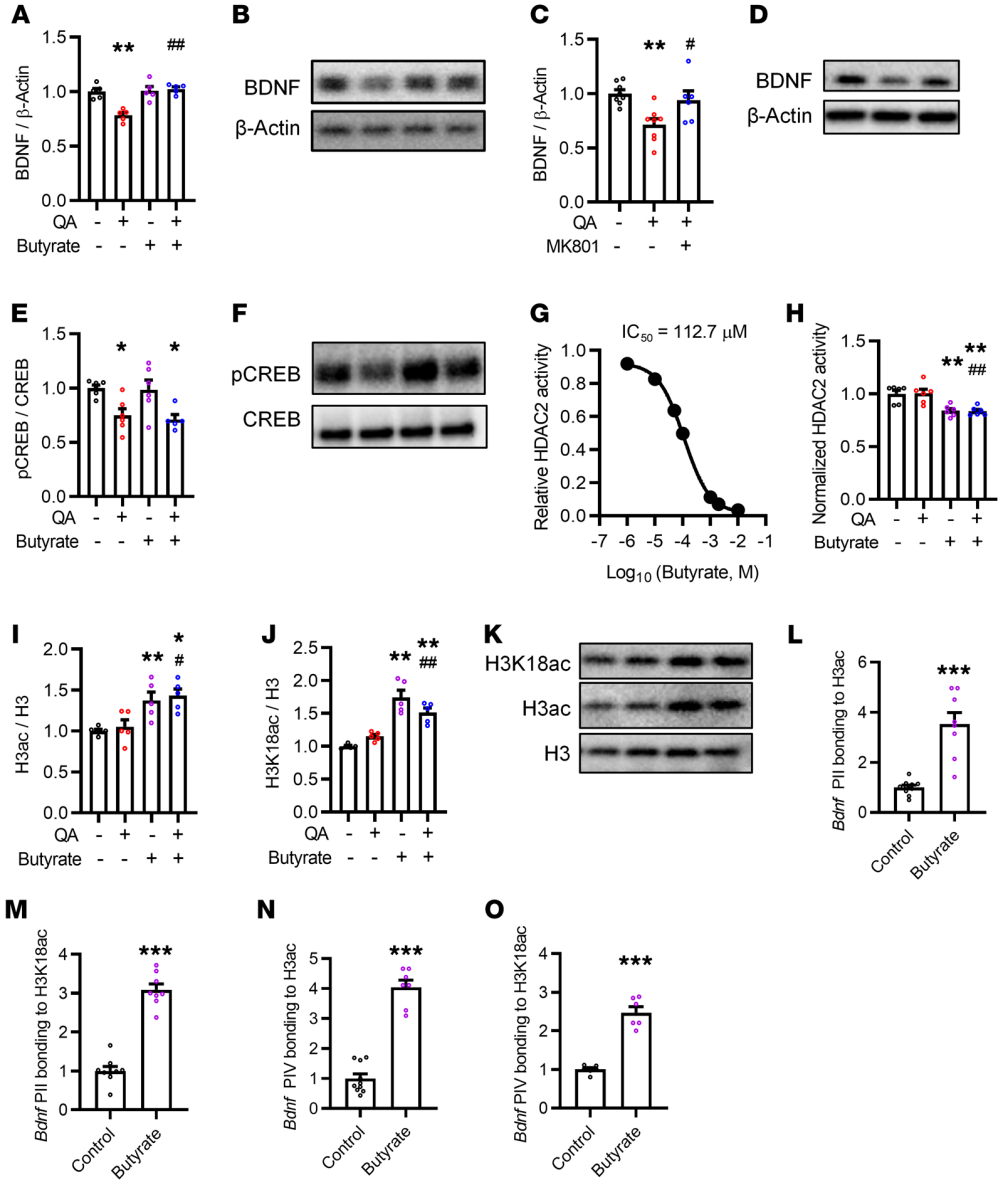
QA reduction of BDNF expression is prevented by butyrate in epigenetically enhanced SH-SY5Y cells. (**A** and **B**) Butyrate reversed QA-reduced BDNF expression in SH-SY5Y cells. (**C** and **D**) MK-801 reversed QA-reduced BDNF expression in SH-SY5Y cells. (**E** and **F**) QA decreased the phosphorylation of CREB at serine 133. (**G**) Dose-response curve for butyrate in the inhibition of HDAC2 enzymatic activity. (**H**) Relative HDAC2 activity of SH-SY5Y cells after treatment with butyrate for 24 hours. (**I**) Butyrate increased the acetylation of histone H3 in SH-SY5Y cells with or without QA exposure. (**J** and **K**) Butyrate increased the acetylation of histone H3 at lysine 18 (H3K18ac) in SH-SY5Y cells with or without QA exposure, as determined by Western blotting. Data indicate the mean ± SEM. *P < 0.05 and **P < 0.01 versus the control group; ^#^*P* < 0.05 and ^##^*P* < 0.01 versus the QA group, by 1-way ANOVA with Tukey’s multiple-comparison test. (**L**–**O**) ChIP analysis showed that butyrate increased the levels of *Bdnf* promoters in the PII region binding to H3ac (**L**) and H3K18ac (**M**) and in the PIV region binding to H3ac (**N**) and H3K18ac (**O**). ***P < 0.001, by Student’s *t* test.

BDNF plays an important role in regulating synaptogenesis (17). Next, we examined whether QA and butyrate could regulate BDNF levels in SH-SY5Y cells. We found that QA decreased BDNF, an action that was prevented by butyrate (*P* < 0.01; Figure 7, A and B). MK-801, an antagonist of the glutamate receptor (*N*-methyl-d-aspartate receptor, NMDAR), blocked QA-induced BDNF reduction (*P* < 0.05; Figure 7, C and D), suggesting that QA reduced BDNF expression via the glutamate NMDA receptor (NMDAR). QA also downregulated the phosphorylation of cAMP response element–binding protein (p-CREB), a BDNF transcription factor. However, butyrate did not affect CREB expression (Figure 7, E and F), indicating that the prevention of BDNF reduction by butyrate was independent of CREB.

Previously, it was reported that BDNF expression is regulated by HDAC2 (31). Using the HDAC2 kinetic assay kit, we found that butyrate inhibited the enzymatic activity of recombinant HDAC2 with the half-maximal inhibitory concentration (IC_50_) at 112.70 μM, which is more potent than that of other SCFAs (propionate and acetate) (Figure 7G and Supplemental Figure 5, A–E). Butyrate significantly inhibited HDAC2 enzymatic activity in SH-SY5Y cells treated with QA (*P* < 0.01; Figure 7H), however, QA did not affect HDAC2 enzymatic activity of expression (Supplemental Figure 5, F–H). It is reported that histone modification H3 and H4 acetylation (H3ac and H4ac, respectively) increase BDNF expression through HDAC inhibition (32). Here, we examined whether butyrate, as an HDAC2 inhibitor, enhances BDNF expression by epigenetic regulation. First, we found that butyrate increased the acetylation of histone H3 (H3ac, *P* < 0.01; Figure 7, I and K), but not histone H4 (H4ac, *P* = 0.67; Supplemental Figure 5, I and K) in SH-SY5Y cells treated or not with QA. Next, butyrate also increased histone H3K18 acetylation (H3K18ac, *P* < 0.01; Figure 7, J and K), a marker enriched at gene promoters and associated with active sites in neurons (33). However, butyrate did not affect H4K8 acetylation (H4K8ac, *P* = 0.52; Supplemental Figure 5, J and K). To determine whether the increase in H3 acetylation is involved in regulating BDNF expression, we assayed the levels of H3ac and H3K18ac at the BDNF promoters PII, PIV and PVI by ChIP. We found that butyrate increased H3ac and H3K18ac binding to BDNF promoters at the PII (both *P* < 0.01; Figure 7, L and M) and PIV (both *P* < 0.01; Figure 7, N and O) regions, without affecting the PVI region (Supplemental Figure 5, L and M). Overall, these results indicate that butyrate as an HDAC2 inhibitor increased histone H3K18 acetylation and its binding on BDNF promoters, thereby rescuing QA-induced BDNF reduction in neurons.

## Discussion

**Figure 8:**
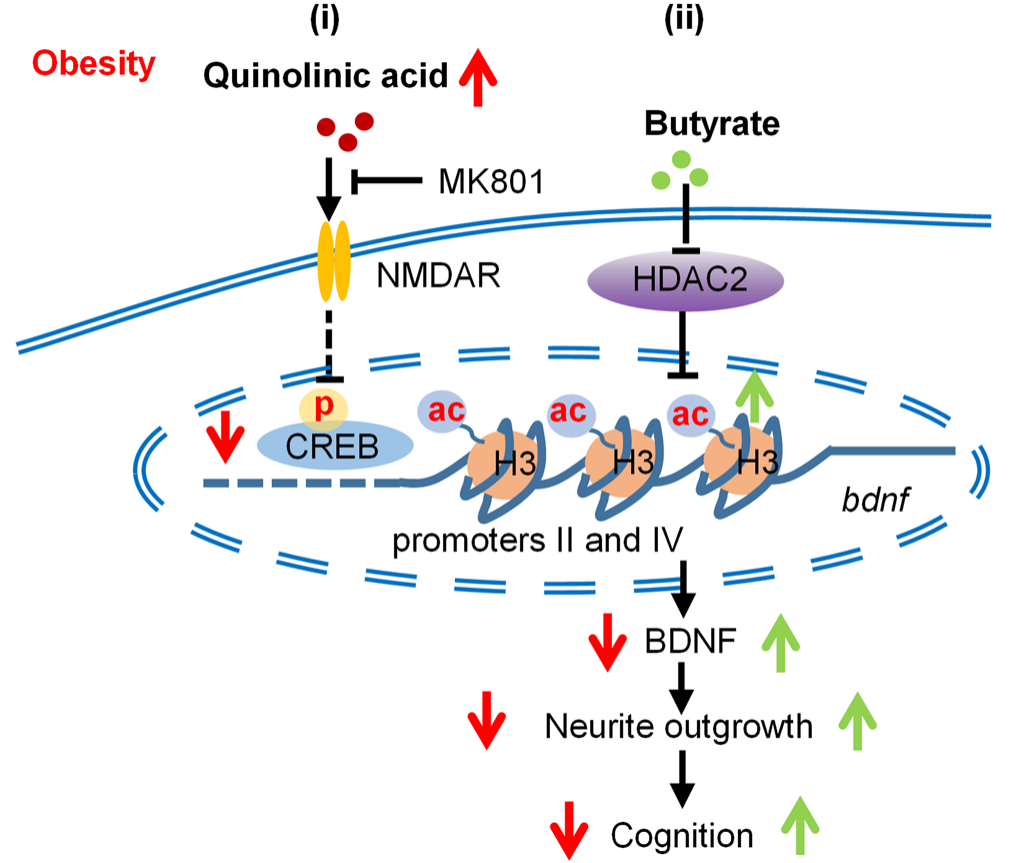
Molecular mechanism of butyrate in preventing QA-induced cognitive and synaptogenesis impairments. (i) In obesity, QA is increased, which activates the NMDAR, downregulates the expression of BDNF and the transcription factor p-CREB, and induces neurite deficits and cognitive decline. (ii) Butyrate inhibits the enzymatic activity of HDAC2 to increase histone H3 acetylation and binding to the BDNF promoters PII and PIV, which prevents QA-induced BDNF reduction and reverses neurite and cognitive impairments.

In this comprehensive assessment, we report that increased QA was associated with cognitive impairment in both individuals with obesity and obese rodents. Our study provides the first evidence to our knowledge that butyrate prevents the cognitive and neurite impairments associated with increased QA in rodents and *C. elegans* and in in vitro cell models relevant to obesity. As an HDAC2 inhibitor, butyrate promoted histone H3K18 hyperacetylation and epigenetic regulation of the BDNF PII and PIV promoter regions to increase BDNF expression, thereby promoting synaptogenesis in neurons (Figure 8).

Excessive QA is a metabolite of the kynurenine pathway in macrophages and microglia (6) and is considered to be involved in the pathogenesis of neurodegenerative diseases, including AD (16). Obesity is a chronic, low-grade systemic and neuroinflammatory disease with activation of macrophages and microglia (4) and is associated with brain structure alterations and cognitive impairment (2, 34). Here, we found that QA levels increased in the serum of Chinese individuals with obesity. This finding aligns with a previous report that QA-related enzyme genes are elevated in the omental adipose tissue of White women with obesity (7). Expanding on the above results showing that increased QA in the blood and peripheral tissue in individuals with obesity, we demonstrated that QA levels of diet-induced obese mice were increased in the frontal cortex, the brain region critical for cognitive function. In *C. elegans*, exposure to QA resulted in cognitive impairment, including impairment of long-term learning and memory and short-term memory. It is reported that QA is increased in peripheral monocytes of patients with AD (35). In postmortem brain tissue of these patients, QA and its synthesis enzyme indoleamine 2,3 dioxygenase are significantly more abundant than in control individuals (36). Therefore, the increased QA in obesity may act as a neuroinflammatory neurotoxin involved in cascade events leading to neurodegeneration.

We found that serum QA levels were negatively correlated with the total cognition score of the RBANS, particularly with regard to the delayed memory index. Interestingly, the delayed memory index was most affected in patients with AD with mild cognitive impairment (37). Previous imaging studies have reported that reduced GMV or cortical thickness is associated with cognitive decline in healthy or aging populations (38). Also, frontal GMV reduction is most affected, including the middle, inferior, and superior frontal gyri in patients with AD or in patients with mild cognitive impairment who developed AD (39). Our results support previous findings that cortical GMV was affected mainly in the frontal cortices of individuals with obesity. Overconsumption of dietary fat and obesity are considered risk factors for AD (40). Importantly, we found that in high-fat diet–induced obese mice, QA levels were increased in the frontal cortex and were negatively associated with recognition memory and spatial working memory in the temporal order and Y maze tests. Therefore, elevated QA levels may contribute to the reduction in frontal cortical volume and poor cognitive performance observed in obesity.

Previous Voxel-based morphometric studies have demonstrated gray matter atrophy from antemortem MRI corresponding to Braak’s stages of AD (41–43). In the present study, GMV reduction in individuals with obesity was observed in the frontal lobe, parietal lobe, temporal lobe, limbic lobe, and insula cortex, which are brain regions involved in Braak stages III–VI (the frontal and parietal lobes belong to Braak stages V/VI; the temporal lobes, limbic lobe, and insula belong to Braak stages III/IV) (44). However, without a postmortem brain tissue study, we cannot verify the corresponding pathological Braak’s stage of the individuals with obesity. It was reported that synapse loss is an early event contributing to the cognitive decline process; however, synaptic losses showed no relationship to Braak stages (45). We and others have found that obese mice have impaired synapses in the cortical and hippocampal areas of the brain (46–50). The present study revealed that cortical GMV was affected in individuals with obesity. A previous study reported that impaired executive function and memory in patients with type 2 diabetes correlated predominantly with reduced gray matter density in the orbital and prefrontal cortices and temporal (middle gyrus, parahippocampus, and uncus) regions (51). Therefore, these data indicate that metabolic disorder–related synapse loss can occur early in the cortex and limbic system and contribute to cognitive decline.

In the present study, the cortical reductions were sometimes observed only in 1 hemisphere. The prevalence of asymmetrical cerebral alterations has been extensively documented in the brains of patients with AD (52), schizophrenia (53), autism (54), anxiety disorders (55), or even type II diabetes (56). In the present study, GMV was reduced in the right temporal lobe of patients with obesity. Both left and right temporal lobes are involved in cognitive function, but in different cognitive properties. For example, the right superior temporal gyrus is involved in executive attention (57). Consistently, in the present study, the volume of the right superior temporal gyrus was decreased in patients with obesity, who had a decline in their attention index. Furthermore, it was reported that reduced GMV in the right middle temporal gyrus is associated with early mild cognitive impairment (58) and abnormal attention (59). In addition, the changes observed in the MRI in the obese human brain may be reversible, not permanent. Previous studies reported that the GMV reduction in the human brain of obese individuals was reversible after bariatric surgery, including gastric bypass surgery and sleeve gastrectomy surgery (60–65). For instance, 1 month after bariatric surgery, gray matter densities were increased in multiple brain regions, including the inferior frontal gyrus, the superior frontal gyrus, the inferior temporal gyrus, the middle temporal gyrus, and the insula (60, 61). GMV in the inferior frontal gyrus showed significant increases 3 months after laparoscopic sleeve gastrectomy surgery compared with before surgery and 1 month after surgery, indicating sustained structural recovery of the brain following bariatric surgery (61). Furthermore, the prompt and sustained improvements in cognitive function including memory, executive function, and attention were observed in patients after bariatric surgery (66).

Dopaminergic and glutamatergic neurotransmission systems are essential for the regulation of cognitive behaviors and functions (28, 29). For example, it is reported that working memory is impaired in the spatial delayed response task of common marmosets with a loss of dopaminergic neurons (67). In the present study, QA led to neurite impairment of CEP dopaminergic neurons in the *C. elegans* BZ555 strain. CEP neurons send axon-like projections to neurons in the nerve ring, the largest collection of ganglia in the worm (68). Therefore, QA-induced damage of neurites of CEP dopaminergic neurons may further mediate the dysregulation of other neurotransmitter systems, such as glutamatergic transmission. Indeed, research shows that dopamine-D1R inhibits glutamate ionotropic NMDAR in neurons (69). Furthermore, it is reported that 20 mM QA triggers neurodegeneration via activation of the NMDAR subunit NR1 in *C. elegans* (70). Here, expanding the postsynaptic NMDAR effect, we found that 20 mM QA decreased expression of the presynaptic glutamate transporter eat-4, which is responsible for glutamate reuptake and prevention of glutamate spillover from the presynapse (71). Therefore, QA overstimulated the glutamate system from presynapse, which may have induced overaccumulation of glutamate in the synapse cleft and overstimulation of the postsynapse NMDAR, leading to the neuroexcitotoxicity and cognition impairment seen in the *C. elegans* model of this study. In addition, BDNF modulated by glutamate and dopamine neurotransmission promotes neurogenesis and learning and memory (72–74). For example, blocking the NMDAR increases BDNF translation and concentration in the hippocampus (72, 73). In the present study, we found that BDNF levels decreased in the frontal cortex of obese mice and in SH-SY5Y cells after exposure to QA. Collectively, these findings suggest that QA-induced dopaminergic and glutamatergic neuron damage and BDNF downregulation are the cellular and molecular mechanisms underlying cognitive decline in obesity.

Butyrate is one of the important SCFAs, the fermentation products of dietary fiber by gut microbiota. Importantly, we found that butyrate supplementation prevented the decline in temporal order memory and Y maze working memory in obese mice and prevented the cognitive decline in *C. elegans* exposed to QA. It is reported that butyric acid–producing bacteria, such as *Clostridium butyricum*, counteract cognitive decline in the vascular dementia mouse model (75). Furthermore, dietary fiber supplementation improves cognition in obese mice (46). Therefore, our findings suggest that butyrate is a possible mediator of butyrate-producing prebiotic or probiotic products that improve cognitive function. Interestingly, we found that butyrate ameliorated QA-induced dopaminergic and glutamatergic neuron damage. Previous research reports that gut microbes modulate dopaminergic and glutamatergic neurotransmission (76, 77). Butyrate-producing microbiota are increased in the gut of mice with higher dopamine levels in the brain (78). These findings collectively suggest that butyrate-induced improvement of dopaminergic and glutamatergic systems plays a vital role in the gut/brain axis mediating cognitive function.

In the present study, butyrate supplementation significantly decreased body weight in obese mice on a high-fat diet. Also, there were negative correlations between body weight and the discrimination index of the temporal order memory test and the alternation triplet of the Y maze spontaneous alternation test. This finding indicates that the butyrate-induced weight loss contributed to cognition improvement. The previous meta-analysis study shows that weight loss is associated with improvements in cognitive function among overweight individuals and people with obesity (79). Weight loss could improve cognition through several mechanisms. First, weight loss attenuates insulin resistance, while insulin resistance is associated with poorer cognitive status (80). Second, weight loss reduces inflammation and oxidative stress, both of which are pathological factors for cognitive decline (81, 82). Our results indicate that butyrate improved cognition and that this improvement was not solely dependent on weight loss. Using body weight as a confounding covariate, we showed that butyrate treatment nonetheless substantially increased the discrimination index in obese mice. Furthermore, our in vitro and *C. elegans* studies showed that butyrate improved neurite outgrowth and cognition (short- and long-term memory), supporting the idea that butyrate may improve cognition irrespective of body weight changes.

Neurite outgrowth and arborization are essential for neural connectivity and cognitive function (9, 10). We showed that butyrate prevented neurite impairment and synaptic spine loss induced by QA in primary frontal cortical and SH-SY5Y cells and in the frontal cortex of obese mice. Neurites and their dendritic spine integrity are essential in synaptic transmission and plasticity, linking their morphophysiology to cognition processes (83). Mushroom spines with large spine heads form strong synaptic connections and have the longest lifetime and are therefore thought to be sites of long-term memory storage (84, 85). Here, we report that butyrate increased the percentage of mushroom spines in the frontal cortex of obese mice.

BDNF positively modulates neurite outgrowth and spine architecture in neurons (86, 87). Altered BDNF and its receptor TrkB significantly impair neurite outgrowth in PC12 neuronal cells (87). Acute BDNF application results in a fast and transient TrkB activation associated with spine head enlargement in neurons (88). In our study, butyrate completely prevented the BDNF reduction induced by QA in SH-SY5Y cells and in the frontal cortex of obese mice. Therefore, an increase in BDNF induced by butyrate may protect synaptic structure and plasticity, which QA impairs. Although the exact mechanism is not fully understood, we showed 2 pathways involved: glutamate/NMDAR/p-CREB/BDNF and HDAC2 epigenetic regulation of BDNF. The previous study reported that QA decreases cortical and striatal BDNF after QA-induced brain lesions in rats (17), although the mechanism was not investigated. At the neuronal level, our study showed that QA reduced BDNF, accompanied by a reduction of its transcription factor p-CREB. The QA-induced reduction of BDNF is primarily attributable to overstimulation of the glutamate system, as MK-801 blocked the ability of QA to decrease BDNF. Moreover, we found that butyrate, which prevented a reduction of BDNF, was unlikely to target the NMDAR/p-CREB/BDNF pathway, since butyrate did not affect p-CREB levels. Instead, the HDAC2 inhibitor butyrate increased histone H3 acetylation at promoter regions II and IV of the BDNF gene. These findings suggest that butyrate promotes BDNF expression by epigenetic regulation. H3K18 acetylation has been found to be significantly reduced in postmortem brain tissue of patients with AD (89). Here, we report butyrate-induced posttranslational modifications of H3K18 acetylation in SH-SY5Y cells. It is known that histone hypoacetylation is a feature of several neurodegenerative diseases and impairs cognition (61); deciphering specific acetylation sites of butyrate may help in the development of a therapeutic strategy to improve cognitive function.

In summary, we report that QA levels were substantially increased in the serum of individuals with obesity and in the frontal cortex of diet-induced obese mouse brain. In both conditions, QA levels were negatively correlated with a decline in cognition accompanied by frontal cortical thinning in individuals with obesity and neurite impairment in diet-induced obese mice. Interestingly, butyrate prevented cognitive impairments in both obese mice and transgenic *C. elegans* exposed to QA. Furthermore, the cellular study showed that butyrate prevented neurite impairment in obese mice and dendritic spine loss in primary cortical neurons exposed to QA. Our experimental results identified that butyrate promoted histone H3K18 hyperacetylation and epigenetic regulation of the BDNF PII and PIV promoter regions to increase BDNF expression. These findings collectively suggest that, in humans, rodents, *C. elegans*, and in vitro cell models, increased QA was associated with cognition decline and neurite lesions relevant to obesity, whereas butyrate alleviated the cognitive impairment induced by higher QA levels or obesity.

## Methods

A detailed description of the experimental procedures is provided in Supplemental Methods.

### Human study design and participants.

Eight-four patients with type 2 diabetes, 42 individuals with obesity (BMI = 30.87 ± 3.02 kg/m^2^), and 42 age- and sex-matched lean controls (BMI = 21.68 ± 1.18 kg/m^2^) were recruited at the First Affiliated Hospital of Liaoning University of Traditional Chinese Medicine. Cognitive function was assessed using RBANS (90). Sixty-three individuals without type 2 diabetes, 40 individuals with obesity (BMI = 29.59 ± 1.63 kg/m^2^), and 23 lean controls (BMI = 22.07 ± 1.43 kg/m^2^) were recruited at the Affiliated Hospital of Xuzhou Medical University, and all were seeking medical attention for symptoms of dizziness, headache, nausea, or vomiting. Cognitive functions were assessed using the MoCA test (91). For both of the above studies, strict exclusion criteria were applied to eliminate the possible influence of confounding factors including: (a) alcohol abuse and smoking, (b) a history of other brain diseases (brain tumor, epilepsy, encephalitis), (c) a current or past history of psychiatric or neurological disease, and (d) taking any current medication that could affect the brain (e.g., antipsychotic drug). The main physical and metabolic characteristics of the study participants are presented in Supplemental Table 1. According to previous studies, in the Chinese population, individuals with a BMI of 28 kg/m^2^ or higher are considered obese (92). In the present study, serum QA levels were determined by ELISA (see details in the Supplemental Methods). In addition, MRIs of 40 individuals — 19 individuals with obesity and 21 nonobese individuals, matched by age and sex (obese, BMI = 30.17 ± 1.99 kg/m^2^; nonobese, BMI = 21.80 ± 1.37 kg/m^2^) — were obtained from the Affiliated Hospital of Xuzhou Medical University. These 40 individuals sought medical attention for the same reasons described above and were subjected to the same exclusion criteria mentioned above. The individuals’ physical and metabolic characteristics are presented in Supplemental Table 2. MRI data were acquired from imaging studies performed on a 3T MR scanner (Discovery 750w, GE Healthcare). T1-weighted scans were preprocessed and analyzed using voxel-based morphometry (VBM) within Statistical Parametric Mapping (SPM) 12 software program (The Wellcome Centre for Human Neuroimaging, London, United Kingdom) running in MATLAB (MathWorks) (93). After realignment, coregistering, normalization, and smooth correction, the differences between individuals with obesity and lean controls were assessed using independent samples *t* tests between the 2 groups to create a group difference map with SPM12 software. The brain regions with decreased GMV in the obese group are labeled in blue on the map.

### Animals and treatments.

Forty C57Bl/6 J male mice (7 weeks old) were divided into 4 groups (*n* = 10): mice in the control group received a lab chow diet (5% fat by weight); mice in the butyrate group received a lab chow diet mixed with butyrate (5% w/w); mice assigned to the obese group received a high-fat diet (31.5% fat by weight); and mice assigned to the obese B group received a high-fat diet mixed with butyrate (5% w/w). After 15 weeks of the interventions, temporal order memory test and Y maze alteration tests were performed. Frontal cortex tissues were collected for further analysis, including Golgi-Cox staining and BDNF measurements (see details in the Supplemental Methods).

### C. elegans cultures and treatment.

All strains of *C. elegans* were obtained from the Caenorhabditis Genetics Center (CGC) at the University of Minnesota (Minneapolis, Minnesota, USA) and maintained according to standard protocols as previously described (94). Behavior tests, including long-term or short-term learning and memory tests and assessments of body-bend rates, pharyngeal pumping rates, and synchronized lifespans were performed (detailed in the Supplemental Methods).

### SH-SY5Y cell cultures and treatments.

After differentiation, SH-SY5Y cells (American Type Culture Collection [ATCC], CRL-2266) were treated with a medium containing different concentrations of QA and/or sodium butyrate. Cell viability was assessed by MTT assay. Neurite length was measured in real time using Incucyte Zoom and analyzed with Neuro Track software (Sartorius). The treated cells were used to examine butyrate inhibition of HDAC2 by enzymatic activity assay and epigenetic regulation of BDNF by ChIP and Western blotting (detailed in the Supplemental Methods).

### Primary cortical neuron cultures and treatment.

Cultured frontal cortical neurons were maintained at 37°C in a humidified 5% CO_2_ incubator for 7 days in vitro (DIV 7) prior to treatments. The neurons were stained with primary antibodies MAP2 (M4403-2ML, MilliporeSigma) or phalloidin followed by the neurite and spine morphology assay (detailed in the Supplemental Methods).

### Statistics.

SPSS (version 21, IBM) was used for statistical analysis. Quantitative data are expressed as the mean ± SEM. Comparisons between 2 groups were performed using a 2-tailed Student’s *t* test. Multiple comparisons were made using 1-way ANOVA), Tukey’s test was used for multiple comparisons, and Dunnett’s test was used to compare multiple samples with the same control. Non-normal data were analyzed with a nonparametric test and are expressed as the median ± quartile deviation. Data on neurite length acquired in real time were analyzed using a repeated-measures ANOVA, and Tukey’s test was used for multiple comparisons with the control. Survival data were analyzed by log-rank (Mantel-Cox) test and multiple comparisons of survival data followed by Bonferroni’s correction. *P* values of less than 0.05 were considered statistically significant.

### Study approval.

All study protocols involving mice were approved by the IACUC of Xuzhou Medical University and conducted in accordance with the Chinese Council on Animal Care Guidelines for the care and use of animals. Human QA and cognition measurements were approved by the Research Ethics Committee of the First Affiliated Hospital of Liaoning University of Traditional Chinese Medicine. Written informed consent was obtained from the patients before participation in the study. The study involving analysis of human MRI scanned images was approved by the University of the Clinical Research Ethics Committee of the Affiliated Hospital of Xuzhou Medical University. The Committee granted a waiver of informed consent because all imaging data were retrospectively collected with anonymity.

## Author contributions

KZ, YY, and XFH designed the research study. XG and M Zheng acquired and analyzed data. MH, XF, DG, SL, LW, JZ, LG, PZ, YX, WP, M Zhou, LZ, and RT performed the experiments. XG, YY, and XFH wrote the manuscript.

## Supplementary Material

Supplemental data

## Figures and Tables

**Table 1:**
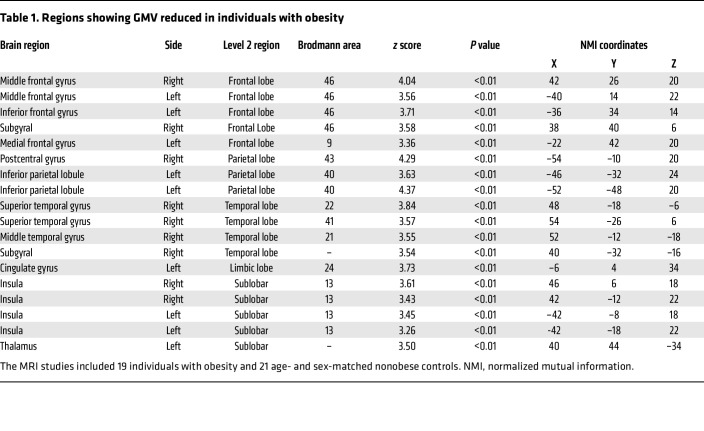
Regions showing GMV reduced in individuals with obesity

## Version 1 02/15/2023

Electronic publication
