# Supplementary material for: Butyrate ameliorates quinolinic acid–induced cognitive decline in obesity models
Source: J Clin Invest. 2023 Feb 15;133(4):e154612. doi: 10.1172/JCI154612 (PMC9927952; doi:10.1172/JCI154612)
Supplement: Supplemental data [file jci-133-154612-s223.pdf]

Supplemental material

Butyrate ameliorates quinolinic acid-induced cognitive decline in obesity models

Xing Ge[1#], Mingxuan Zheng[1#], Minmin Hu[1#], Xiaoli Fang[2#], Deqin Geng[2], Sha Liu[2], Li Wang[3], Jun Zhang[3], Li Guan[4], Peng Zheng[5], Yuanyi Xie[5], Wei Pan[1], Menglu Zhou[1], Limian Zhou[1], Renxian Tang[1], Kuiyang Zheng*[1], Yinghua Yu*[1], Xu-Feng Huang*[1, 5]

Affiliations

[1]Jiangsu Key Laboratory of Immunity and Metabolism, Department of Pathogen Biology and Immunology, Xuzhou Medical University, Xuzhou, Jiangsu 221004, China;

[2]Department of Neurology, Affiliated Hospital of Xuzhou Medical University, Jiangsu 221006, China;

[3]Affiliated Hospital of Liaoning University of Traditional Chinese Medicine, Shenyang, Liaoning 110032, China;

[4]The Second Affiliated Hospital of Liaoning University of Traditional Chinese Medicine, Shenyang, Liaoning 110034, China;

[5]Illawarra Health and Medical Research Institute (IHMRI) and School of Medicine, Indigenous, and Health, University of Wollongong, NSW 2522, Australia.

[#]Contributed equally to this paper

*Correspondence

Professor Kuiyang Zheng, MD, PhD, Jiangsu Key Laboratory for Immunity and Metabolism, Department of Pathogen Biology and Immunology, Xuzhou Medical University, Jiangsu 221004, China. Email: zky02@163.com

Professor Yinghua Yu, MD, PhD, Jiangsu Key Laboratory for Immunity and Metabolism, Department of Pathogen Biology and Immunology, Xuzhou Medical University, Jiangsu 221004, China. Email: yinghuayu@xzhmu.edu.cn; yinghua@uow.edu.au; ORCID: orcid.org/0000-0003-2508-7512

Distinguished Professor Xu-Feng Huang, MD, PhD, D.Sc, Illawarra Health and Medical Research Institute and School of Medicine, Indigenous, and Health, University of Wollongong, NSW2522, Australia. Email: xhuang@uow.edu.au; ORCID: orcid.org/0000000258952253

Supplemental Data

Supplemental Figures 1-5

Supplemental Tables 1-6

Supplemental Methods

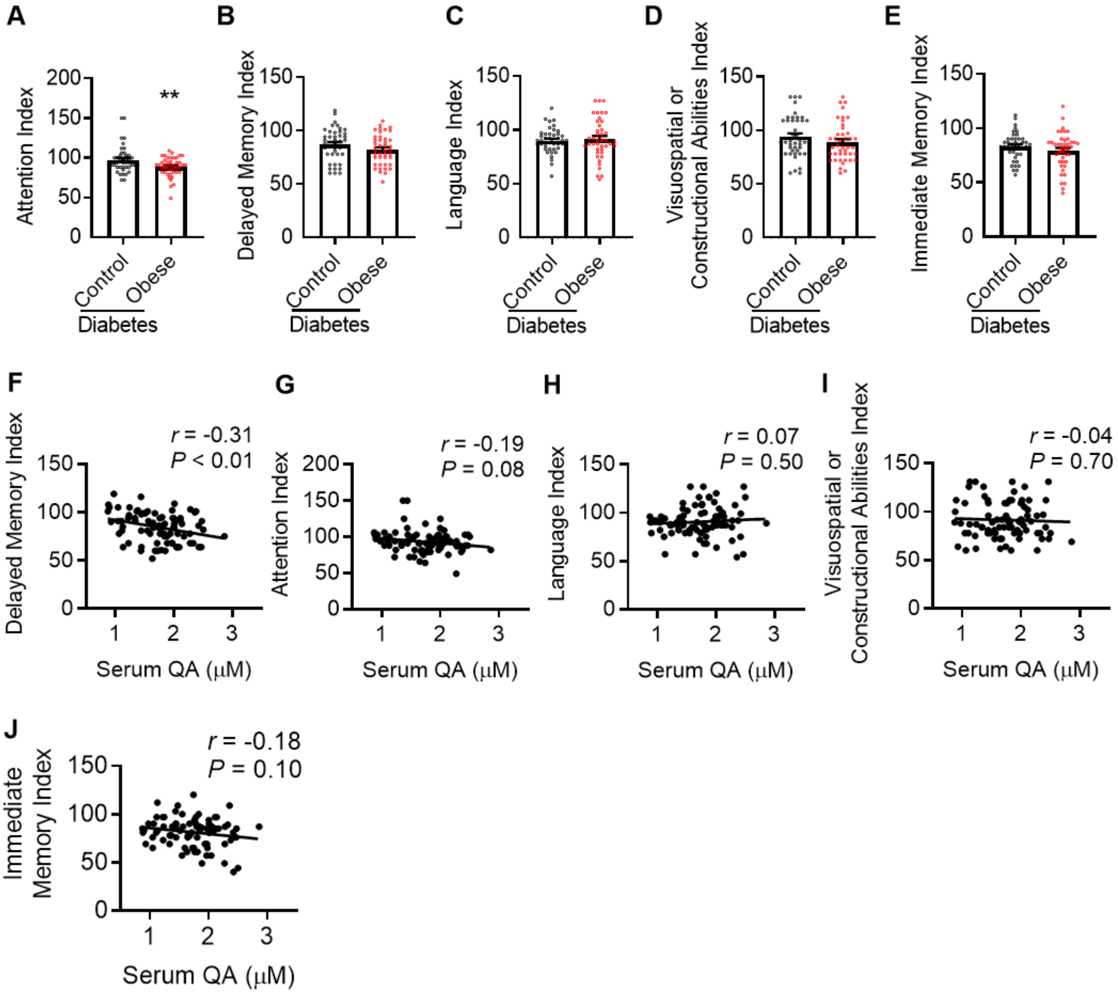

Supplemental Figure 1. The cognitive function was declined and negatively correlated with serum quinolinic acid in obese subjects with type 2 diabetes. (A-E) The attention index (A), delayed memory index (B), language index (C), visuospatial or constructional abilities index (D) and immediate memory index (E) in obese subjects. **(F-J)** Serum quinolinic acid was negatively correlated with delayed memory index (F), but not correlated with attention index (G), language index (H), visuospatial or constructional abilities index (I) and immediate memory index (J). **$P < 0.01$ comparing with control group, Student's *t* test. QA, quinolinic acid.

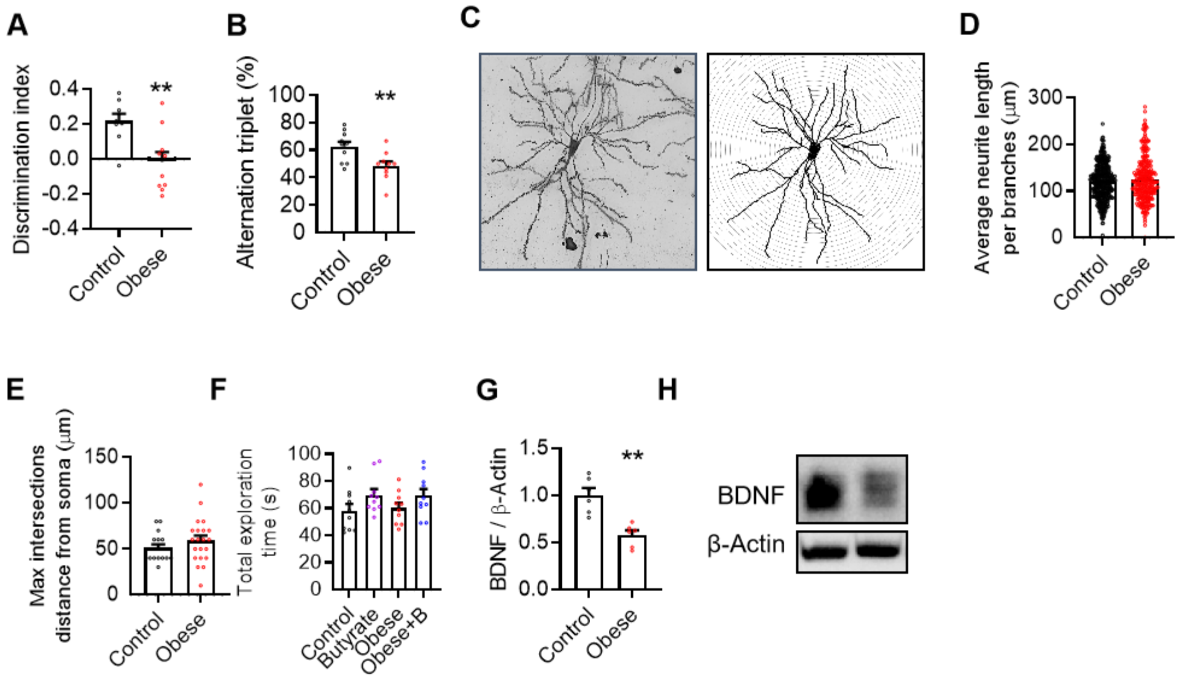

Supplemental Figure 2. Obese mice had a cognitive decline and synaptogenesis impairment. (A) The discrimination index in temporal order memory tests was significantly decreased in obese mice compared to the control mice. **(B)** The alternation triplet in Y maze tests was significantly decreased in obese mice compared to the control mice. **(C)** Representative images of Golgi-Cox-impregnated photomicrographs and reconstructions of dendrites of neurons. **(D)** The average neurite length per branch in the frontal cortex. **(E)** The max intersections from the soma in the frontal cortex. **(F)** The total exploration time tested by temporal order experiments. Analyzed by 1-way ANOVA. **(G and H)** The BDNF expression was decreased in the frontal cortex in obese mice. **$P < 0.01$ comparing with control group, Student's t test. Obese, high-fat diet-induced obese mice. Obese-B, mice fed with a high-fat diet mixed with butyrate.

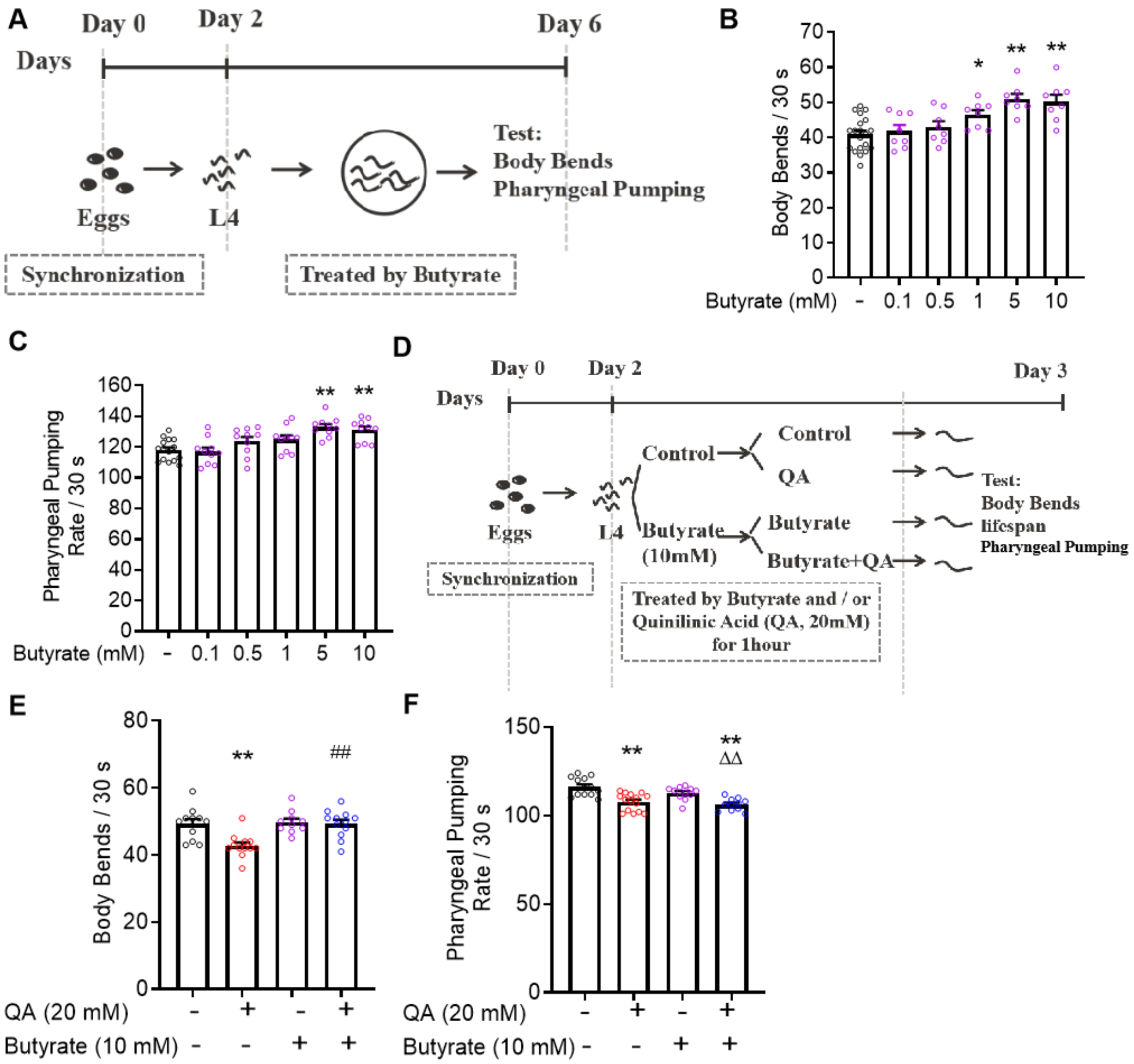

Supplemental Figure 3. The effect of butyrate on the behavior of C. elegans. (A) Experimental procedure of the butyrate on the behaviors of C. elegans. **(B-C)** Body bends / 30 s (B) and pharyngeal pumping rate (C) of C. elegans treated with butyrate for 4 days. $*P < 0.05$, $**P < 0.01$ comparing with control group, 1-way ANOVA, Dunnett's multiple-comparisons test. **(D)** Experimental procedure to examine the effect of butyrate and QA on the behavior of C. elegans. **(E)** Body bends of C. elegans treated with QA (20 mM) and/or butyrate (10 mM) for 1 hour. **(F)** The pharyngeal pumping rate of C. elegans treated with QA (20 mM) and/or butyrate (10 mM) for 1 hour. Mean ± SEM. $**P < 0.01$ comparing with control group, $^{\#\#}P < 0.01$ comparing with QA group, $^{\Delta\Delta}P < 0.01$ compared with butyrate group, 1-way ANOVA, Tukey's multiple-comparisons test. QA, quinolinic acid.

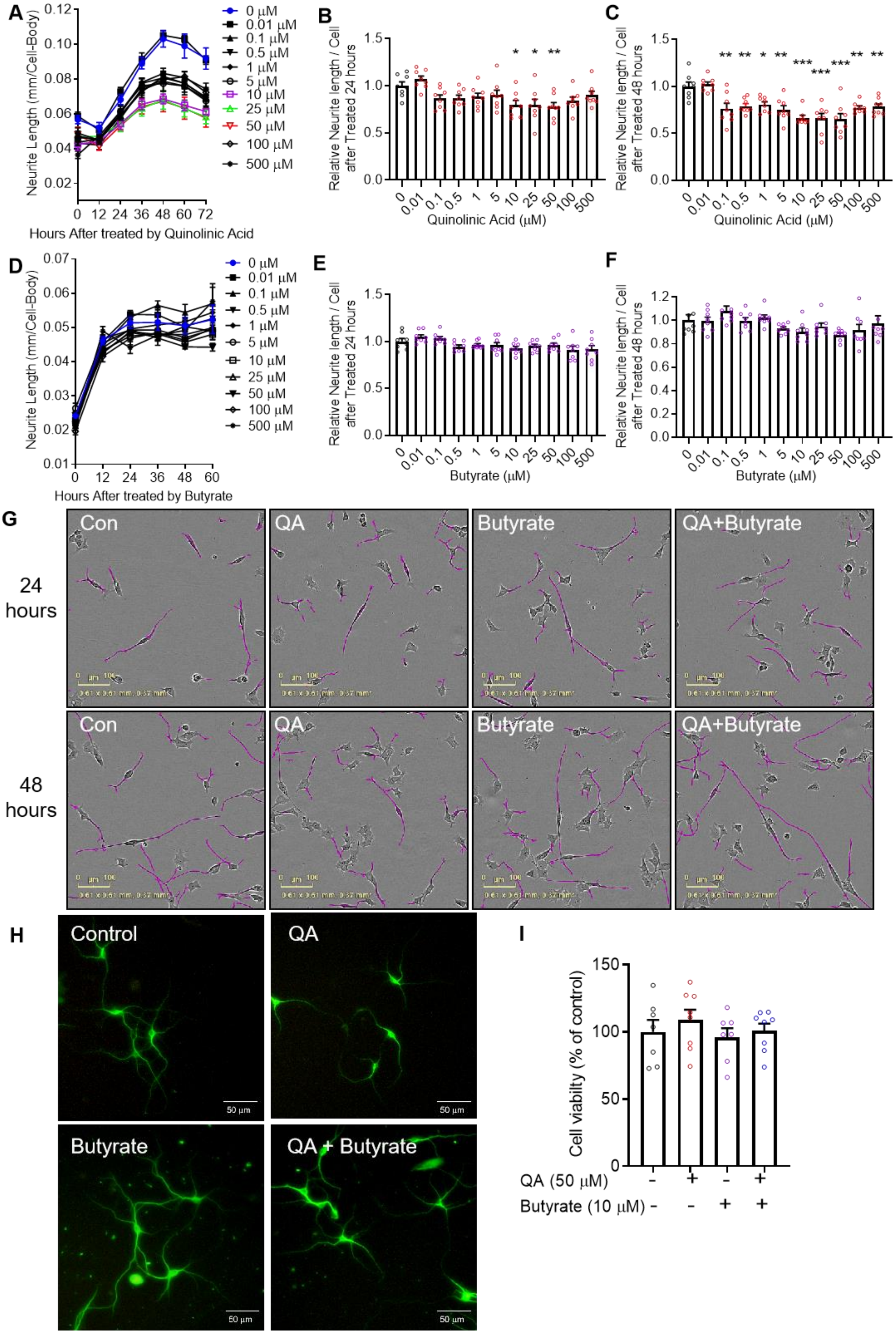

A
Neurite Length (mm/Cell-Body)
Hours After treated by Quinolinic Acid
0 μM
0.01 μM
0.1 μM
0.5 μM
1 μM
5 μM
10 μM
25 μM
50 μM
100 μM
500 μM
B
Relative Neurite length / Cell after Treated 24 hours
Quinolinic Acid (μM)
C
Relative Neurite length / Cell after Treated 48 hours
Quinolinic Acid (μM)
D
Neurite Length (mm/Cell-Body)
Hours After treated by Butyrate
E
Relative Neurite length / Cell after Treated 24 hours
Butyrate (μM)
F
Relative Neurite length / Cell after Treated 48 hours
Butyrate (μM)
G
24 hours
48 hours
Con
QA
Butyrate
QA+Butyrate
H
Control
QA
Butyrate
QA + Butyrate
50 μm
I
Cell viability (% of control)
QA (50 μM)
Butyrate (10 μM)

Supplemental Figure 4. The effects of quinolinic acid and butyrate on neurite outgrowth. SH-SY5Y cells were treated with various concentrations of quinolinic acid or sodium butyrate (0, 0.01, 0.1, 0.5, 1, 10, 25, 50, 100, 500 µM) after differentiated by 10 µM retinoic acid for 24 hours. **(A)** Quinolinic acid decreased the neurite length of SH-SY5Y (n = 8 run in triplicate). **(B-C)** Neurite length was reduced after quinolinic acid administration at 10, 25, 50 µM for 24 hours (B) and after quinolinic acid administration (0.1-500 µM) for 48 hours (C). **(D)** Butyrate treatment had no significant effect on neurite outgrowth of SH-SY5Y cells (n = 8 run in triplicate). **(E-F)** Neurite length of SH-SY5Y after treated by butyrate for 24 hours (E) and for 48 hours (F). **(G)** Representative images of SH-SY5Y cells treated by quinolinic acid and/or butyrate. **(H)** Primary frontal cortical neurons (DIV 7) treated by QA (50 µM) and/or butyrate (10 µM) for 24 hours, stained with MAP2 antibody, and imaged with immunofluorescence microscope. **(I)** Cell viability after quinolinic acid and butyrate treatment for 24h assayed by MTT. *$P < 0.05$, **$P < 0.01$, ***$P < 0.001$ comparing with control group, 1-way ANOVA, Dunnett's multiple-comparisons test. QA, quinolinic acid.

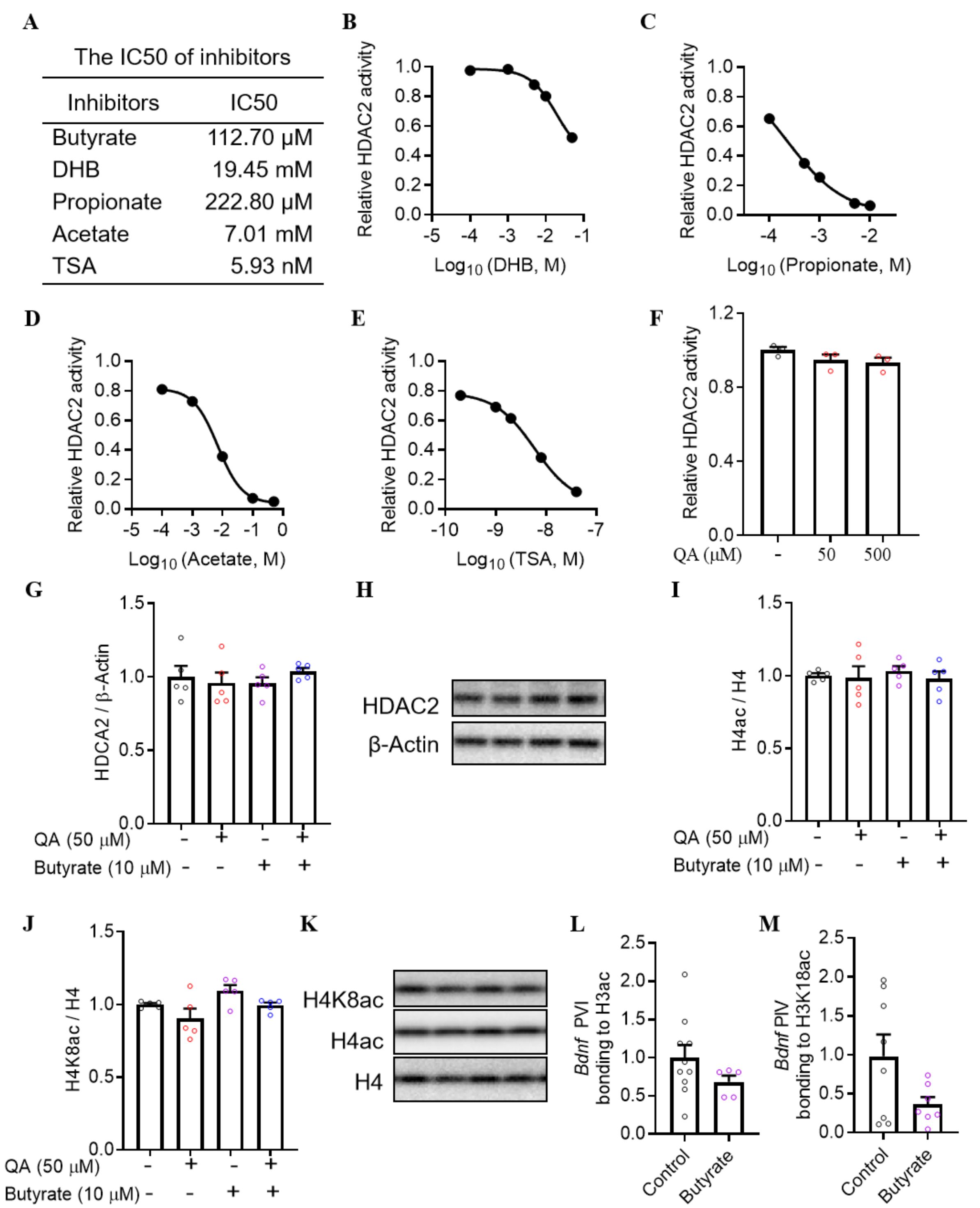

Supplemental Figure 5. The effects of butyrate, other HDAC inhibitors and quinolinic acid on HDAC2 enzyme activity, and effects of butyrate and QA on HDAC2 protein level, H4 acetylation. (A) The IC_{50} of HDAC inhibitors of HDAC2. **(B-E)** The dose-response curve of D-β-hydroxybutyrate (DHB, has a similar chemical structure with butyrate, B), propionate (one of SCFAs, C), acetate (one of SCFAs, D) and Trichostatin A (TSA, a potent inhibitor of HDAC, E) in inhibition of HDAC2 enzyme activity. **(F)** quinolinic acid treatment did not affect HDAC2 enzyme activity. (A-F) HDAC2 enzyme activity was examined by which HDAC2 (1.0 ng/well) was incubated (37°C) with 10 μM fluor de Lys®-Green substrate and HDAC

inhibitors or quinolinic acid. Reactions were stopped after 60 min with Fluor de Lys® Developer, and fluorescence was measured (Ex. 485 nm, Em. 528 nm). **(G-H)** The HDAC2 protein level in SH-SY5Y cells treated by QA and/or butyrate for 24 hours by the Western bolt. **(I-K)** Butyrate did not affect histone H4 acetylation (H4ac) and H4K8 acetylation (H4K8ac) in SH-SY5Y cells treated with or without quinolinic acid. Mean ± SEM. Analyzed by 1-way ANOVA. **(L-M)** Butyrate did not affect H3ac (L) and H3K18ac (M) binding to BDNF promoters PVI. QA, quinolinic acid. Analyzed by Student's *t* test.

Supplemental Table 1 Demographic characteristics and general metabolic profile of the obese and non-obese groups in cognition study.

Variable	Control	Obese	$t/\chi^2/Z$	*P*
**Cognition study of diabetes (Type 2)**	**n = 42**	**n = 42**		
Age (years)*	56.00 ± 16.50	57.00 ± 13.50	-1.33	0.18
BMI (kg/m^2)	21.68 ± 1.18	30.87 ± 3.02	-17.78	**<0.01**
SBP (mm Hg)*	130.00 ± 20.00	130.00 ± 21.30	-0.71	0.48
DBP (mm Hg)*	80.00 ± 10.00	80.00 ± 10.00	-1.20	0.23
Fasting blood glucose (mmol/L)*	7.67 ± 3.40	9.80 ± 3.52	-1.34	0.18
Fasting inulin (μU/ml)	5.81 ± 0.69	11.33 ± 3.51	-7.74	**<0.01**
HOMA-IR*	1.61 ± 1.03	4.17 ± 3.65	-4.87	**<0.01**
Fasting C peptide (ng/ml)	0.95 ± 0.88	1.36 ± 1.04	-1.84	0.07
HbA1C (%)*	7.95 ± 3.15	7.80 ± 3.08	-0.40	0.69
C-reactive protein (mg/L)*	2.60 ± 6.02	6.30 ± 6.34	-0.36	0.72
TC (mmol/L)*	4.55 ± 1.06	5.34 ± 1.37	-2.95	**<0.01**
TG (mmol/L)*	1.18 ± 0.94	1.93 ± 1.91	-3.07	**<0.01**
HDL (mmol/L)*	1.09 ± 1.00	1.14 ± 0.89	-0.61	0.55
LDL (mmol/L)*	2.73 ± 0.84	3.40 ± 1.33	-2.47	**0.01**
ALT (U/L)*	21.00 ± 19.50	23.00 ± 27.30	-1.35	0.18
AST (U/L)*	18.00 ± 7.50	21.50 ± 11.00	-1.20	0.23
ApoA1 (g/L)	1.23 ± 0.27	1.26 ± 0.23	-0.51	0.61
ApoB1 (g/L)*	0.90 ± 0.21	1.02 ± 0.29	-2.63	**<0.01**
UA (μmol/L)	294.59 ± 97.30	335.62 ± 128.62	-1.59	0.12
White blood cells (%)	6.17 ± 1.52	6.47 ± 1.64	-0.85	0.40
Leukocyte (%)*	1.87 ± 1.00	1.97 ± 0.68	-0.54	0.59
Monocytes (%)*	0.35 ± 0.13	0.37 ± 0.12	-0.86	0.39
**Cognition study of non-diabetes**	**n = 40**	**n = 23**		
Age (years)	55.15 ± 10.33	59.00 ± 11.68	-1.36	0.18
BMI (kg/m^2)	22.07 ±1.43	29.59 ± 1.63	-19.10	**<0.01**
SBP (mm Hg)	123.20 ± 25.20	137.48 ± 17.50	-2.40	**0.02**
DBP (mm Hg)*	80.00 ± 14.00	82 ± 12.00	-1.67	0.09
Fasting blood glucose (mmol/L)	4.96 ± 1.50	5.23 ± 1.43	-0.67	0.51
HbA1C (%)	6.51 ± 2.78	6.07 ± 1.08	0.52	0.61
C-reactive protein (mg/L)	1.10 ± 1.95	4.42 ± 6.32	-2.38	**0.02**
TC (mmol/L)	4.27 ± 0.89	3.98 ± 0.91	1.20	0.24
TG (mmol/L)	1.32 ± 0.52	1.70 ± 0.71	-2.37	**0.02**
HDL (mmol/L)	1.16 ± 0.23	0.96 ± 0.24	3.16	**<0.01**
LDL (mmol/L)	2.48 ± 0.75	2.47 ± 0.63	0.03	0.98
ApoA1 (g/L)	1.10 ± 0.11	1.00 ± 0.13	1.77	0.10
ApoB1 (g/L)	0.79 ± 0.23	0.77 ± 0.09	0.21	0.83
White blood cells (10^9/L)	5.45 ± 1.60	6.24 ± 1.55	-1.77	0.08
Neutrocyte (%)	57.86 ± 12.06	60.97 ± 9.72	-0.97	0.34

Leukocyte (%)	31.64 ± 10.93	30.48 ± 8.82	0.40	0.69
Monocytes (%)	6.27 ± 1.08	6.27 ± 1.83	-0.01	0.99
Eosinophils (%)	2.51 ± 2.06	2.26 ± 1.40	0.47	0.64
Basophils (%)	0.57 ± 0.28	0.54 ± 0.21	0.43	0.67

BMI, body mass index. SBP, systolic blood pressure. DBP, diastolic blood pressure. HbA1C, glycosylated hemoglobin. TC, total cholesterol. TG, triglyceride. HDL, high-density lipoprotein. LDL, low-density lipoprotein. ALT, alanine aminotransferase. AST, aspartate aminotransferase. ApoA1, Apolipoprotein A1. ApoB1, apolipoprotein B1. HOMA-IR, homeostasis model assessment of insulin resistance. UA, uric acid. *Non-normal data expressed as Median ± Quartile Deviation, and analyzed by nonparametric test.

Supplemental Table 2 Demographic characteristics and general metabolic profile of the obese and control groups in MRI study.

Variable	Control (n = 21)	Obese (n = 19)	$t/\chi^2/Z$	*P*
Age (years)	53.43 ± 8.54	50.84 ± 14.08	-0.69	0.49
BMI (kg/m^2)	21.80 ± 1.37	30.17 ± 1.99	15.23	**<0.01**
SBP (mm Hg)*	140.00 ± 37.00	140.00 ± 24.00	-0.36	0.72
DBP (mm Hg)*	80.00 ± 29.00	80.00 ± 11.00	-0.97	0.33
Fasting blood glucose (mmol/L)	5.57 ± 2.83	5.47 ± 1.27	0.13	0.90
TC (mmol/L)	4.04 ± 0.77	4.59 ± 1.04	-1.84	0.07
TG (mmol/L)*	1.20 ± 0.66	2.15 ± 2.01	-2.84	**<0.01**
HDL (mmol/L)	1.09 ± 0.27	0.96 ± 0.19	1.66	0.11
LDL (mmol/L)	2.26 ± 0.65	2.52 ± 0.75	-1.16	0.26
ApoA1 (g/L)	1.29 ± 0.21	1.31 ± 0.21	-0.29	0.77
ApoB1 (g/L)	0.79 ± 0.12	0.97 ± 0.18	-3.22	**<0.01**
White blood cells (10^9/L)	5.16 ± 1.42	5.94 ± 1.35	-1.70	0.10
Neutrocyte (%)*	61.60 ± 18.10	52.80 ± 14.90	-0.02	0.99
Leukocyte (%)	34.48 ± 9.51	35.01 ± 7.59	-0.19	0.85
Monocytes (%)*	6.50 ± 2.90	5.80 ± 2.10	-0.55	0.58
Eosinophils (%)*	1.70 ± 2.70	2.20 ± 1.90	-0.54	0.59
Basophils (%)*	0.50 ± 0.40	0.35 ± 0.30	-2.00	**<0.05**

BMI, body mass index. SBP, systolic blood pressure. DBP, diastolic blood pressure. TC, total cholesterol. TG, triglyceride. HDL, high-density lipoprotein. LDL, low-density lipoprotein. ApoA1, Apolipoprotein A1. ApoB1, apolipoprotein B1. *Non-normal data expressed as Median ± Quartile Deviation, and analyzed by nonparametric test.

Supplemental Table 3 The correlation between metabolic profiles and serum QA

Variable	Correlation with QA	
	r	*P*
**Cognition study of type 2 diabetes**		
BMI (kg/m^2)	0.54	**<0.01**
Fasting blood glucose (mmol/L)	-0.05	0.73
Fasting inulin (μU/ml)	0.32	**0.02**
HOMA-IR	0.21	0.13
HbA1C (%)	0.09	0.44
C-reactive protein (mg/L)	0.09	0.69
TC (mmol/L)	0.05	0.67
TG (mmol/L)	0.14	0.22
HDL (mmol/L)	-0.03	0.77
LDL (mmol/L)	0.08	0.51
ApoA1 (g/L)	0.01	0.92
ApoB1 (g/L)	0.02	0.84
**Cognition study of non-diabetes**		
BMI (kg/m^2)	0.39	**<0.01**
Fasting blood glucose (mmol/L)	0.08	0.57
HbA1C (%)	-0.13	0.50
C-reactive protein (mg/L)	-0.11	0.54
TC (mmol/L)	-0.12	0.36
TG (mmol/L)	-0.08	0.55
HDL (mmol/L)	-0.07	0.61
LDL (mmol/L)	-0.01	0.96
ApoA1 (g/L)	-0.36	0.15
ApoB1 (g/L)	-0.12	0.66

QA, quinolinic acid. BMI, body mass index. HOMA-IR, homeostasis model assessment of insulin resistance. HbA1C, glycosylated hemoglobin. TC, total cholesterol. TG, triglyceride. HDL, high-density lipoprotein. LDL, low-density lipoprotein. ApoA1, Apolipoprotein A1. ApoB1, apolipoprotein B1.

Supplemental Table 4 General metabolic profile of the obese and control mice

Variable	Control	Obese	*t*	*P*
**General characteristics**				
Weight (g)	27.71 ± 1.38	36.46 ± 2.14	-8.41	**<0.01**
Subcutaneous fat mass (g)	0.30 ± 0.06	1.90 ± 0.38	-10.14	**<0.01**
Epididymis fat mass (g)	0.34 ± 0.08	1.41 ± 0.19	-12.60	**<0.01**
Brown fat mass (g)	0.12 ± 0.03	0.31 ± 0.18	-2.48	0.05
**General metabolic profile**				
Fasting blood glucose (mmol/L)	5.48 ± 0.58	6.60 ± 2.98	-0.90	0.41
Fasting inulin (μU/ml)	16.68 ± 13.33	29.47 ± 14.41	-1.60	0.14
HOMA-IR	3.92 ± 2.84	9.67 ± 7.78	-1.70	0.14
1hPPG (mmol/L)	12.30 ± 2.49	20.08 ± 3.41	-4.52	**<0.01**
2hPPG (mmol/L)	7.68 ± 2.35	14.05 ± 4.96	-2.84	**0.02**
TC (mmol/L)	3.58 ± 0.40	5.90 ± 0.97	-5.42	**<0.01**
TG (mmol/L)	0.70 ± 0.14	0.53 ± 0.16	1.81	0.10
HDL (mmol/L)	1.99 ± 0.25	3.59 ± 0.62	-5.83	**<0.01**
LDL (mmol/L)	0.20 ± 0.06	0.58 ± 0.16	-5.36	**<0.01**

HOMA-IR, homeostasis model assessment of insulin resistance. 1hPPG, 1-hour post-load plasma glucose during an oral glucose tolerance test (OGTT). 2hPPG, 2-hour post-load plasma glucose during OGTT. TC, serum total cholesterol. TG, triglyceride. HDL, high-density lipoprotein. LDL, low-density lipoprotein.

Supplemental Table 5 General metabolic profile of obese mice with or without butyrate treatment

Variable	Obese	Obese-B	*t*	*P*
**General characteristics**				
Weight (g)	36.46 ± 2.14	33.03 ± 3.71	2.526	**0.03**
Subcutaneous fat mass (g)	1.90 ± 0.38	0.69 ± 0.44	5.05	**<0.01**
Epididymis fat mass (g)	1.41 ± 0.19	1.27 ± 0.62	0.54	0.60
Brown fat mass (g)	0.31 ± 0.18	0.13 ± 0.03	2.31	0.07
**General metabolic profile**				
Fasting blood glucose (mmol/L)	6.60 ± 2.98	6.20 ± 1.54	0.29	0.78
Fasting inulin (μU/ml)	29.47 ± 14.41	12.43 ± 4.81	2.75	**0.02**
HOMA-IR	9.67 ± 7.78	3.63 ± 2.22	-1.83	0.12
1hPPG (mmol/L)	20.08 ± 3.41	21.87 ± 4.54	-0.77	0.46
2hPPG (mmol/L)	14.05 ± 4.96	17.12 ± 4.05	-1.17	0.27
TC (mmol/L)	5.90 ± 0.97	5.23 ± 0.27	1.62	0.16
TG (mmol/L)	0.85 ± 0.10	1.01 ± 0.26	-1.38	0.20
HDL (mmol/L)	3.59 ± 0.62	3.39 ± 0.20	0.75	0.48
LDL (mmol/L)	0.58 ± 0.16	0.48 ± 0.05	1.55	0.17

HOMA-IR, homeostasis model assessment of insulin resistance. 1hPPG, 1-hour post-load plasma glucose during an oral glucose tolerance test (OGTT). 2hPPG, 2-hour post-load plasma glucose during OGTT. TC, total cholesterol. TG, triglyceride. HDL, high-density lipoprotein. LDL, low-density lipoprotein. Obese-B: obese mice with butyrate treatment.

Supplemental Table 6 The effect of butyrate on the cognition of obese mice tested with analysis of covariance using body weight as confounding covariates.

**Terms**	**Control (n = 21)**	**Obese (n =19)**	***F***	***P***
Discrimination index	-0.02 ± 0.18	0.28 ± 0.17	9.06	**<0.01**
Alternation triplet (%)	50.42 ± 11.88	62.64 ±8.56	3.90	0.07

Supplemental Methods

Cognitive function. The Repeatable Battery for the Assessment of Neuropsychological Status (RBANS) (1) and the Montreal Cognitive Assessment (MoCA) (2) was performed to assess the cognition of obese subjects and control subjects in this study. The RBANS is a neuropsychological test battery comprising 12 subtests (list learning, story memory, figure copy, line orientation, picture naming, semantic fluency, digit span, coding, list recall, list recognition, story recall and figure recall) that are used to calculate index scores for five domains (attention, language, visuospatial or constructional abilities, and immediate and delayed memory). An overall cognition score was derived from the five domains, with higher scores indicating better cognition. The MoCA has a high level of sensitivity and specificity for detecting mild cognitive impairment. It is scored out of 30 with higher scores indicative of better cognition. Education level is accounted for in the scoring, by 1 point added to the score if the number of years of education was lease than 12 years.

ELISA for quinolinic acid in serum and brain tissue. Blood of obese subjects and control subjects was centrifuged at 2000×g for 15 minutes. Serum was collected and stored at -80°C until analysis. The frontal cortex of mice was lysed in lysis buffer containing NP40 (Sigma-Aldrich), protease inhibitor cocktail (Sigma-Aldrich), 1 mM PMSF (Sigma-Aldrich), and 0.5 mM β-glycerophosphate (Sigma-Aldrich). The concentration of quinolinic acid was tested by QA ELISA kit (Gelatins®, Shang Hai, China; Cat.No. JLC3154). Standard and the samples (50 μl) were added to the wells pre-coated with specific antibodies in a 96-well plate, and the assay was conducted as per the manufacturer's instructions. The results were calculated by fitting the

optical density into a four-parametric logistic curve.

Gray matter volume in magnetic resonance imaging (MRI). MRIs of. MRI data of 19 obese patients (BMI = 30.17 ± 1.99 kg/m^2) and 21 age- and sex-matched lean controls (BMI = 21.80 ± 1.37 kg/m^2) were acquired imaging on a 3 T MR scanner (Discovery 750w, GE Healthcare, Waukesha, WI). T1-weighted scans were preprocessed and analyzed using voxel-based morphometry within the Statistical Parametric Mapping (SPM) 12 software (The Wellcome Centre for Human Neuroimaging, London, UK) running in MATLAB (Math Works Inc., Natick, MA, USA) (3). After realignment, coregistration, normalization and smooth correction, the differences of gray matter volumes between obese subjects and healthy controls were examined by the independent samples t-tests between the two groups by SPM12 software. The brain regions with decreased gray matter volume in the obese group were labeled in blue color in a statistical map on 3D-T1 axial and sagittal slice (MNI T1 template available on MRICRON software).

Animals and treatments. Forty C57Bl/6 J male mice (7 weeks old) were obtained from the Experimental Animal Center of Xuzhou Medical University, and housed in environmentally controlled conditions (temperature 22℃, 12 hours light/dark cycle). After one week acclimatization period, the mice were divided into four groups (n=10) received either lab chow or high-fat diet with/without butyrate supplementation for 21 weeks: mice received a lab chow diet as a control group (5% fat by weight) (final body weight = 28.06 ± 1.77 g); mice received with butyrate mixed into lab chow (5% w/w) diet as butyrate group (final body weight = 28.09

± 1.84 g); mice received a high-fat diet (31.5% fat by weight: soybean oil 55 g and lard 260 g per kilogram) as an obese group (final body weight = 37.24 ± 2.36 g); mice received the high-fat diet mixed with butyrate (5% w/w) as an obese-B group (final body weight = 33.70 ± 5.62 g). After the intervention, the temporal order memory test and Y-maze alterations test were performed. Three days after the last test, mice were sacrificed using CO_2 asphyxiation. Frontal cortex tissues were collected and stored in −80°C for further analyses, including Golgi-Cox staining and BDNF measurement.

Behavioral tests of mice. The temporal order memory and Y-maze alterations tests were performed to examine the effects of butyrate on spatial and recognition memory based on methods previously described (4-6). In the temporal order memory test, the discrimination index was calculated as [(Time with the older object-Time with recent object)/Total time with both objects]×100. For the Y-maze alterations test, the alternation was defined as if a mouse enters a different arm of the maze in each of 3 consecutive arm entries. The alternation triplet (%) was calculated as [number of successful alternations/(total number of arms entries-2)×100].

Golgi staining. After perfusion with 10% paraformaldehyde, mice brains were stained by using the Rapid Golgi stain Kit (FD NeuroTechnologies) according to the manufacturer's protocol. (7). Coronal sections (100 μm) of Golgi-stained brains were cut using a cryostat with the temperature set at -18°C. Slices were mounted on microscope slides (Fisherbrand; Fisher Scientific, Waltham, MA). Images were taken using a digital scanning microscope (Olympus VS120). The criteria used to select pyramidal neurons for reconstruction were: (a) full

impregnation of the neurons along the entire length of the dendritic tree; (b) dendrites without significant truncation of branches; and (c) relative isolation from neighboring impregnated neurons, astrocytes, or blood vessels. More than 15 neurons were selected in each group. Each neuron was traced by using the Neuron J algorithm supported by the NIH Fiji software (https ://image j.net/ Fiji) to create images of reconstructions. The neurite length was measured using the ImageJ Software. Sholl analysis was then performed on the reconstructions to measure the number of dendritic processes that cross concentric circles spaced 10 μm apart starting at the center of the soma. The number of intersections at each radius provides a profile of neuronal arborization, which is described by metrics including sum intersections (the sum number of intersections), the maximum number of intersections (the max of intersections of one circle) and max intersection distance (the distance of max intersections from the soma). For spine analysis, the spine density was analyzed with Fiji software by counting the number of dendritic spines per 10 μm on a 30–50 μm segment of a distal branch. Dendritic spines were classified as thin, mushroom-like and stubby spines (8, 9). Thin spines were defined as dendritic protrusions shorter than 5 μm and lacking a clearly defined head. Mushroom-like spines were defined as having a clear round stubby head. Stubby spines were defined as spines without a neck. There were 4 mice in each group for Golgi staining.

C. elegans culture and treatment. All strains of C. elegans were obtained from the Caenorhabditis Genetics Center (CGC, University of Minnesota, Minneapolis, MN, USA) and maintained according to standard protocols as previously described (10). BZ555 (dat-1p::GFP), DA1240 (eat-4::GFP+lin-15(+)) and EG1285 (unc-47p::GFP+lin-15(+)) transgenic strains

respectively visualize dopaminergic (11), glutamatergic (12) and GABAergic (13-15) neurons in C. elegans. Neurons in C. elegans were viewed using a 10× objective or 40× oil immersion objective on a DMi8 fluorescence microscope (Leica, Mannheim, Germany). Worms were exposed to quinolinic acid and/or butyrate at the L4 stage. Quinolinic acid and butyrate were mixed into the nematode growth media (NGM), previously seeded with *Escherichia coli* OP50 1 hour before exposing the worms. Subsequently, the worms were washed three times and the analyses were performed.

The behavior of C. elegans. 1) Long-term or short-term learning and memory: C. elegans were trained and tested for either long-term or short-term learning and memory as previously described in positive associative olfactory learning and memory assay (16, 17). Firstly, chemotaxis assays were performed according to previously described methods (18). Briefly, worms were placed at the origin area in test plate, NGM plates with two tested areas, butanone (1 μl 1:10 butanone: ethanol + 1 μl NaN_3) and ethanol control (1 μl ethanol + 1 μl NaN_3). One hour later, the number of worms at the origin area, butanone area and ethanol control area were counted. Chemotaxis index naive (CIN) = (Number of worms at butanone − Number of worms at ethanol control) / (Total number of worms). In long-term learning and memory test, worms were trained by alternation between 30 minutes conditioning cycles (seeded with OP50 *E. coli bacteria* and with 6 μl 10% butanone in ethanol on the lid) and 30 minutes starvation cycles (on NGM plates with no food) for 7 times. After training, chemotaxis assays were performed at 0, 12, 24, 48 hours. Chemotaxis index trained (CIT) = (Number of worms at butanone − Number of worms at ethanol control) / (Total number of worms) was calculated. The long-term

learning index (0 hour) and memory index (12, 24 and 48 hours) were calculated by the formula (long-term index = CIT – CIN). In short-term learning and memory test, worms were trained in NGM conditioning plates (seeded with OP50 *E. coli bacteria* and with 6 μl 10% butanone in ethanol on the lid) for 1 hour. After training, the worms were tested for chemotaxis assays at 0, 0.5, 1, 1.5 and 2 hours. The short-term learning index (0 hour) and memory index (0.5, 1, 1.5 and 2 hours) was calculated by the formula (short-term index = CIT – CIN).

2) Body bends rate assay: The body bends rate assay was conducted following criteria from previous reports (19). Worms were transferred into a bacteria-free NGM plate and allowed to acclimate for 60 seconds. The total number of body bends was quantified during 30 seconds in a stereomicroscope. Each body bending was considered as the change of direction of the worm's head characterized by the presence of the pharyngeal bulb towards the right side.

3) Pharyngeal pumping rate assay: Synchronized worms were raised on OP50-seeded plates and allowed to adapt for 10 minutes at room temperature (approximately 20 °C), and then the pharyngeal pumping rate was counted under a stereomicroscope.

4) Synchronized lifespan assay: C. elegans were treated with quinolinic acid (20 mM) for 1 hour, followed by the administration of 10 mM butyrate or vehicle every day at the L4 stage. The survivals were recorded every day until all of the worms died (n = 125 / treatment).

SH-SY5Y cell culture and treatments. SH-SY5Y cells obtained from the American Type Culture Collection (ATCC, CRL-2266) were grown in Dulbecco's modified Eagle's medium (DMEM)-F12 supplemented with 10% heat-inactivated fetal bovine serum (FBS) and 1% penicillin-streptomycin from Bovogen Biologicals (Victoria, Australia). For differentiation, cells were

seeded in plates coated with MaxGel™ ECM (E0282, Sigma-Aldrich, Syndey). In the following day, the medium was removed and replaced with 10 μM retinoic acid (RA, R2625, Sigma-Aldrich) in DMEM-F12 with 1% FBS and 1% penicillin-streptomycin. Cells were treated with a medium containing different concentrations of quinolinic acid (P63204, Sigma-Aldrich) and/or butyrate (B5887, Sigma-Aldrich) after differentiation. The neurite length was acquired in real-time every 6 hours for 72 hours using IncucyteZoom and analyzed with the Neuro Track software (Sartorius, Michigan). There were four groups in our experiment, including the control group, quinolinic acid treatment group (QA group), butyrate treatment group and quinolinic acid with butyrate treatment group (QA+Butyrate group).

Cell viability assay. Cell viability was assessed by an MTT assay kit (Santa Cru Biotechnology). After treatment, the MTT mixture was loaded to each well, and the plate was further incubated at 37°C for 4 hours. The purple dye was dissolved with dimethyl sulfoxide (DMSO), and absorbance was observed at 570 nm.

HDAC2 enzymatic activity. Inhibition of HDAC2 activity of butyrate, D-β-hydroxybutyrate (DHB), propionate, acetate and trichostatin A (TSA) was tested by HDAC2 Fluorimetric Drug Discovery Kit (BML-AK512, Enabling Discovery in Life Science®). In short, HDAC2 (1.0 ng/well) was incubated (37°C) with 10 μM fluor de Lys®-Green substrate and butyrate and other HDAC inhibitors. Reactions were stopped after 60 minutes with Fluor de Lys® Developer and fluorescence was measured (Ex. 485 nm, Em. 528 nm). Relative HDAC2 activity in SH-SY5Y cells was tested after being treated by butyrate with or with quinolinic

acid for 24 hours. HDAC2 was separated by immunoprecipitation with HDAC2 antibody (Cell Signaling Technology). Separated HDAC2 was incubated (37°C) with 10 μM fluor de Lys®-Green substrate. Reactions were stopped after 60 minutes with Fluor de Lys® Developer, and fluorescence was measured (Ex. 485 nm, Em. 528 nm).

Western blot. Cells or tissues were lysed with lysis buffer containing NP40 (Sigma-Aldrich), Protease Inhibitor Cocktail (Sigma-Aldrich), 1 mM PMSF (Sigma-Aldrich), and 0.5 mM β-glycerophosphate (Sigma-Aldrich). Total protein concentrations were determined by DC-Assay (Bio-Rad, Sydney) and detected with a SpectraMax Plus384 absorbance microplate reader (Molecular Devices, Sunnyvale, CA). Samples were heat-treated in Laemmli buffer at 95°C, loaded to 10% SDS-PAGE gels (Bio-Rad) for fractionation, and then transferred into Immune-Blot TM PVDF membranes (Bio-Rad). The blocking buffer consisted of 5% slim milk in TBST. The membranes were incubated with HDAC2 (5113, Cell Signaling Technology), BDNF (SC-20981, Santa Cruz), CREB (PLA0205, Sigma-Aldrich), pCREB (pSer^{133}, SAB4504375, Sigma-Aldrich), β-actin (AC026, ABclonal), Histone H3ac (ab47915, Abcam), Histone H3K18ac (ab40888, Abcam), Histone H3 (ab1791, Abcam), Histone H4ac (ab177790, Abcam), Histone H4K8ac (ab45166, Abcam) and Histone H4 (ab177840, Abcam) antibodies in Tris Buffered Saline Tween (TBST) containing 1% slim milk at 4°C overnight. Secondary antibodies were anti-rabbit or anti-mouse IgG conjugated with horseradish peroxidase (Santa Cruz Biotechnology). For visualization, we used ECL detection reagents and obtained high-resolution images with Amersham Gel Imager (GE Healthcare life Sciences).

Quantitative real-time PCR (RT-PCR). Total RNA of cells was extracted by Aurum total RNA mini kit (Bio-Rad Laboratories, Hercules, CA). Total RNA reverse transcribed to complementary first strand DNA with a high-capacity cDNA reverse transcription kit (AB Applied Biosystems, California, USA) according to the manufacturer's directions. Quantitative real-time PCR (RT-PCR) was performed using a Lightcycler 480 real-time PCR system (F.Hoffmann-La Roche Ltd, Switzerland). A 20 μl final reaction volume containing cDNA sample and SYBR green I master mix was used to perform the experiment. The primers were as follows: *Hdac2* forward, 5'-GCTATTCCAGAAGATGCTGTTC-3'; *Hdac2* reverse 5'-GTTGCTGAGCTGTTCTGATTTG-3'; *Gapdh* forward, 5'-TGCACCACCAACTGCTTAGC-3'; *Gapdh* reverse, 5'-TGCACCACCAACTGCTTAGC-3'. Briefly, amplification was carried out with 45 cycles of 95°C for 10 seconds, 60°C for 30 seconds and 72°C for 30 seconds. The expression of mRNA was normalized to internal control and a house-keeping gene (GAPDH). The degree of mRNA expression was calculated using the comparative threshold cycle value (Ct) method, using the formula $2^{-\Delta\Delta Ct}$ (where $\Delta\Delta Ct = \Delta Ct$ sample − ΔCt reference).

Chromatin immunoprecipitation (ChIP). SH-SY5Y cells were treated with quinolinic acid (50 μM) and/or butyrate (10 μM) for 1 hour. After treatment, ChIP assays were performed with Histone H3ac (Abcam) antibody using ChIP Assay Kit following the manufacturer's instructions (Beyotime, Shanghai, China). Briefly, SH-SY5Y cells were cross-linked with 1% paraformaldehyde, before lysis with a cold PBS/protease inhibitor. Chromatin was sheared by 10 seconds of sonication interposed with 30-second pauses, repeated 10 times. Immunoprecipitation was performed with 4 μg of H3ac antibody (ab47915, Abcam) or

H3K18ac antibody (ab40888, Abcam) overnight at 4°C. Each IP sample was added to protein G agarose beads for 3 hours incubation at 4 °C. After pull-down, beads were washed 5 times. The immunocomplexes were extracted and processed by reverse cross-linking, proteinase K digestion, and DNA precipitation. PCR analysis was conducted in a real-time PCR system as previously described. The primer sequences for the BDNF promoters were as follows (20): BDNF PII forward 5'-GAGTCCATTCAGCACCTTGGA-3'; BDNF PII reverse 5'-ATCTCAGTGTGAGCCGAACCT-3'; BDNF PIV forward 5'-AAGCATGCAATGCCCTGGAAC-3'; BDNF PIV reverse 5'-TGCCTTGACGTGCGCTGTCAT-3'; BDNF PVI forward 5'-GTGTTTAAGGCAGCAGAGTAAACC-3'; BDNF PVI reverse 5'-CCATCCCAGCACCCAAGTTCT-3'.

Primary prefrontal cortical neurons culture. Cultured prefrontal cortical neurons were harvested from C57Bl mice within 3 postnatal days. Briefly, prefrontal cortical neurons were gently dissociated with a plastic pipette after digestion with 0.5% trypsin (GIBCO, LosAngeles) at 37 °C for 30 minutes. Neurons were cultured in a neurobasal medium (GIBCO) containing B27 supplement (GIBCO) and 20 mM glutamine (Sigma Aldrich). After 24 hours of culture, 5-fluoro-2'-deoxyuridine (Sigma Aldrich) was added at a final concentration of 10 μM to repress the growth of glial cells. Cultures were maintained at 37°C in a humidified 5% CO_2 incubator for 7 days in vitro (DIV 7) prior to treatments.

Immunofluorescence assay of the neurite. Primary prefrontal cortical neurons were grown to

approximately 70% confluence on glass coverslips and treated with quinolinic acid and/or butyrate for 24 hours. Then neurons were fixed in 4% formaldehyde for 15 minutes. Neurons were washed in phosphate buffer solution (PBS), and permeabilized with 0.3% Triton X-100 in PBS for 10 minutes. After blocking with 5% normal donkey serum for 1 hour at room temperature, primary antibodies of MAP2 (M4403-2ML, Sigma-Aldrich) were applied in 1% donkey serum in PBS at 4°C overnight. This was followed by incubation in a secondary antibody Alexa Fluor 488-conjugated donkey anti-mouse IgG (Invitrogen, Carlsbad, CA) at room temperature for 2 hours. Cells were viewed using 40× or 63× oil immersion objective on a DMI6500B confocal microscope (Leica, Mannheim, Germany). The neurite length was measured using the ImageJ Software. Neuronal morphology was profiled by Sholl analysis, which quantifies intersections of neurites against the radial distance from the soma center (21). The number of intersections of concentric circles and neurites was counted every 10 µm with a maximum radius of 400 µm. Sholl analysis was performed by the Sholl plugin of ImageJ software. The number of intersections at each radius provides a profile of neuronal arborization, which is described by metrics including sum intersections (the sum number of intersections), max intersection distance (the distance of max intersections from the soma), and the maximum number of intersections (the max of intersections from one circle).

Spine morphology assay. Primary prefrontal cortical neurons were used for spine morphology study after being cultured for 17 days in vitro (DIV 17). The procedure was similar to the immunofluorescence assay. After blocking, neurons were incubated with Alexa Fluor TM 568 Phalloidin (A12380, Invitrogen) for 1 hour and washed with PBS. Neurons were viewed using

a 63× oil immersion objective on a DMI6500B confocal microscope (Leica, Mannheim, Germany). The number of synaptic spines was measured using ImageJ Software.

References

1. Randolph C, Tierney MC, Mohr E, and Chase TN. The Repeatable Battery for the Assessment of Neuropsychological Status (RBANS): preliminary clinical validity. *J Clin Exp Neuropsychol.* 1998;20(3):310-9.
2. Shi MY, Ma CC, Chen FF, Zhou XY, Li X, Tang CX, et al. Possible role of glial cell line-derived neurotrophic factor for predicting cognitive impairment in Parkinson's disease: a case-control study. *Neural Regen Res.* 2021;16(5):885-92.
3. Dake MD, De Marco M, Blackburn DJ, Wilkinson ID, Remes A, Liu Y, et al. Obesity and Brain Vulnerability in Normal and Abnormal Aging: A Multimodal MRI Study. *J Alzheimers Dis Rep.* 2021;5(1):65-77.
4. Yau SY, Lee TH, Formolo DA, Lee WL, Li LC, Siu PM, et al. Effects of Maternal Voluntary Wheel Running During Pregnancy on Adult Hippocampal Neurogenesis, Temporal Order Memory, and Depression-Like Behavior in Adult Female and Male Offspring. *Front Neurosci.* 2019;13:470.
5. Barker GR, Bird F, Alexander V, and Warburton EC. Recognition memory for objects, place, and temporal order: a disconnection analysis of the role of the medial prefrontal cortex and perirhinal cortex. *J Neurosci.* 2007;27(11):2948-57.
6. Borbely E, Payrits M, Hunyady A, Mezo G, and Pinter E. Important regulatory function of transient receptor potential ankyrin 1 receptors in age-related learning and memory alterations of mice. *Geroscience.* 2019;41(5):643-54.
7. Graff J, Joseph NF, Horn ME, Samiei A, Meng J, Seo J, et al. Epigenetic priming of memory updating during reconsolidation to attenuate remote fear memories. *Cell.* 2014;156(1-2):261-76.
8. Liu D, Tang H, Li XY, Deng MF, Wei N, Wang X, et al. Targeting the HDAC2/HNF-4A/miR-101b/AMPK Pathway Rescues Tauopathy and Dendritic Abnormalities in Alzheimer's Disease. *Mol Ther.* 2017;25(3):752-64.
9. Du H, Chen X, Zhang L, Liu Y, Zhan C, Chen J, et al. Experimental Autoimmune Prostatitis Induces Learning-Memory Impairment and Structural Neuroplastic Changes in Mice. *Cell Mol Neurobiol.* 2020;40(1):99-111.
10. Brenner S. The genetics of Caenorhabditis elegans. *Genetics.* 1974;77(1):71-94.
11. Maduro M, and Pilgrim D. Conservation of function and expression of unc-119 from two Caenorhabditis species despite divergence of non-coding DNA. *Gene.* 1996;183(1-2):77-85.
12. White JG, Southgate E, Thomson JN, and Brenner S. The structure of the nervous system of the nematode Caenorhabditis elegans. *Philos Trans R Soc Lond B Biol Sci.* 1986;314(1165):1-340.
13. Lee RY, Sawin ER, Chalfie M, Horvitz HR, and Avery L. EAT-4, a homolog of a mammalian sodium-dependent inorganic phosphate cotransporter, is necessary for glutamatergic neurotransmission in caenorhabditis elegans. *J Neurosci.* 1999;19(1):159-67.
14. Li P, Xu T, Wu S, Lei L, and He D. Chronic exposure to graphene-based nanomaterials induces behavioral deficits and neural damage in Caenorhabditis elegans. *J Appl Toxicol.* 2017;37(10):1140-50.
15. Nass R, Miller DM, and Blakely RD. C. elegans: a novel pharmacogenetic model to study Parkinson's

disease. *Parkinsonism Relat Disord.* 2001;7(3):185-91.

16. Kauffman AL, Ashraf JM, Corces-Zimmerman MR, Landis JN, and Murphy CT. Insulin signaling and dietary restriction differentially influence the decline of learning and memory with age. *PLoS Biol.* 2010;8(5):e1000372.

17. Kauffman A, Parsons L, Stein G, Wills A, Kaletsky R, and Murphy C. C. elegans positive butanone learning, short-term, and long-term associative memory assays. *J Vis Exp.* 2011(49).

18. Block F, Kunkel M, and Schwarz M. Quinolinic acid lesion of the striatum induces impairment in spatial learning and motor performance in rats. *Neurosci Lett.* 1993;149(2):126-8.

19. Colonnello A, Aguilera-Portillo G, Rubio-Lopez LC, Robles-Banuelos B, Rangel-Lopez E, Cortez-Nunez S, et al. Comparing the Neuroprotective Effects of Caffeic Acid in Rat Cortical Slices and Caenorhabditis elegans: Involvement of Nrf2 and SKN-1 Signaling Pathways. *Neurotox Res.* 2020;37(2):326-37.

20. He DY, Neasta J, and Ron D. Epigenetic regulation of BDNF expression via the scaffolding protein RACK1. *J Biol Chem.* 2010;285(25):19043-50.

21. Sholl DA. Dendritic organization in the neurons of the visual and motor cortices of the cat. *J Anat.* 1953;87(4):387-406.
